# Analysis of early-flowering genes at barley chromosome 2H expands the repertoire of mutant alleles at the *Mat*-*c* locus

**DOI:** 10.1007/s00299-019-02472-4

**Published:** 2019-09-20

**Authors:** Izabela Matyszczak, Marta Tominska, Shakhira Zakhrabekova, Christoph Dockter, Mats Hansson

**Affiliations:** 1 Carlsberg Research Laboratory, J.C. Jacobsens Gade 4, 1799 Copenhagen V, Denmark; 2 Department of Plant Physiology, Faculty of Biology and Environment Protection, University of Silesia, 40-032 Katowice, Poland; 3 Department of Biology, Lund University, Sölvegatan 35, SE-22362 Lund, Sweden

**Keywords:** *CENTRORADIALIS*, Earliness, *Hordeum vulgare*, *Mat*-*b*, *TERMINAL FLOWER1*, *TFL1*

## Abstract

**Key message:**

Analyses of barley *mat*-*c* loss of function mutants reveal deletions, splice-site mutations and nonsynonymous substitutions in a key gene regulating early flowering.

**Abstract:**

Optimal timing of flowering is critical for reproductive success and crop yield improvement. Several major quantitative trait loci for flowering time variation have been identified in barley. In the present study, we analyzed two near-isogenic lines, BW507 and BW508, which were reported to carry two independent early-flowering mutant loci, *mat*-*b.7* and *mat*-*c.19*, respectively. Both introgression segments are co-localized in the pericentromeric region of chromosome 2H. We mapped the mutation in BW507 to a 31 Mbp interval on chromosome 2HL and concluded that BW507 has a deletion of *Mat*-*c*, which is an ortholog of *Antirrhinum majus CENTRORADIALIS* (*AmCEN*) and *Arabidopsis thaliana TERMINAL FLOWER1* (*AtTFL1*). Contrary to previous reports, our data showed that both BW507 and BW508 are *Mat*-*c* deficient and none of them are *mat*-*b.7* derived. This work complements previous studies by identifying the uncharacterized *mat*-*c.19* mutant and seven additional *mat*-*c* mutants. Moreover, we explored the X-ray structure of AtTFL1 for prediction of the functional effects of nonsynonymous substitutions caused by mutations in *Mat*-*c*.

**Electronic supplementary material:**

The online version of this article (10.1007/s00299-019-02472-4) contains supplementary material, which is available to authorized users.

## Introduction

Floral induction is the change from vegetative to reproductive growth, a key event in the life cycle of flowering plants. Several pathways are known to regulate flowering in the model plant *Arabidopsis thaliana*, including the photoperiod, the vernalization, the autonomous and the gibberellin pathways (Boss et al. 2004). Floral induction is largely dependent on the action of the *FLOWERING LOCUS T* (*FT*) gene product, which plays an essential role in the integration of all exogenous and endogenous inputs that determine the onset of flowering (Kardailsky et al. 1999; Kobayashi et al. 1999; Lifschitz et al. 2006; Turck et al. 2008). It has been shown that *FT* genes are highly conserved and have universal floral promoting functions and that misexpression of *FT* severely influences flowering time (Kardailsky et al. 1999; Kobayashi et al. 1999; Lifschitz et al. 2006; Turck et al. 2008). FT belongs to the phosphatidylethanolamine-binding protein (PEBP) family, which also includes TERMINAL FLOWER1 (TFL1) (Chardon and Damerval 2005; Faure et al. 2007), named CENTRORADIALIS (CEN) in *Antirrhinum majus* (Bradley et al. 1996). TFL1 controls flowering time and inflorescence architecture (Alvarez et al. 1992; Shannon and Meeks-Wagner 1991). Loss-of-function mutations in *TFL1* accelerate flowering whereas overexpression significantly delays flowering. Therefore, *TFL1* is believed to negatively regulate flowering (Hanzawa et al. 2005; Ratcliffe et al. 1998; Simon and Coupland 1996). The barley ortholog of *TFL1*, *HvCEN*, has been identified as a candidate underlying a QTL (Quantitative Trait Locus) that contributed to the differentiation of winter and spring barley growth habit (Comadran et al. 2012). Resequencing of *HvCEN* from a panel of spring and winter barley accessions revealed two haplotypes discriminated by a single Pro-135-Ala amino acid substitution (Comadran et al. 2012). All the winter accessions included proline at position 135, whereas alanine was specific to accessions with a spring growth habit.

*FT* encodes a mobile protein (florigen) that moves from leaves to the shoot apical meristem (SAM) (Corbesier et al. 2007; Jaeger and Wigge 2007; Tamaki et al. 2007) where it creates a complex with the basic leucine zipper (bZIP) transcription factor, FLOWERING LOCUS D (FD), to activate expression of floral meristem identity genes such as *APETALA1* (*AP1*) in *Arabidopsis* (Abe et al. 2005; Wigge et al. 2005), *VERNALIZATION1* (*VRN1*) in wheat (*Triticum aestivum* L.) (Li and Dubcovsky 2008) and *FRUITFULL2* (*FUL2*) in rice (*Oryza sativa*) (Tsuji et al. 2011). The rice and wheat ‘florigen activation complex’ (FAC) additionally includes a 14-3-3 protein (Li et al. 2015; Taoka et al. 2011). The expression of meristem identity genes in *Arabidopsis* is antagonized by TFL1 activity through a TFL1-FD complex which inhibits the switch to reproductive growth (Hanano and Goto 2011).

Despite the opposing functions, FT and TFL1 proteins are highly similar (Hanzawa et al. 2005; Ho and Weigel 2014). A comparison of *Arabidopsis* FT and TFL1 sequences revealed 78% protein identity (Ho and Weigel 2014). The X-ray structures of *Arabidopsis* FT and TFL1, and *A. major* CENTRORADIALIS have been solved (Ahn et al. 2006; Banfield and Brady 2000). As expected from the primary sequence similarities, the fold topologies are highly comparable (Ahn et al. 2006; Ho and Weigel 2014). The potential ligand-binding pocket and a divergent external loop were identified as critical for functional specificity of FT and TFL1 (Ahn et al. 2006; Hanzawa et al. 2005). Interestingly, modifications of the protein surface charge were shown to be largely sufficient to convert FT into a complete TFL1 mimic (Ho and Weigel 2014). Structural modeling of the TFL1 mimic suggested consequences for the docking of an unknown ligand (Ho and Weigel 2014) rather than docking of known FT and TFL1 interacting proteins like FD and 14-3-3 (Ahn et al. 2006; Taoka et al. 2011; Wigge et al. 2005). The members of the TCP (TEOSINTE BRANCHED1, CYCLOIDE, PCF) family of transcription factors were anticipated as potential candidates for mediating antagonistic functions of FT and TFL1 (Cubas et al. 1999; Ho and Weigel 2014). However, the mechanism remains unclear.

Understanding the genetic basis of flowering time control is of general importance for the improvement of small-grain crop cultivars worldwide, as flowering time is a key factor for adaptation to natural and agricultural settings and directly influence yield (Turner et al. 2005). Flowering time in barley is a complex trait that exhibits almost continuous variation (Cockram et al. 2007). A number of QTL studies have identified mainly three classes of genes regulating flowering time in barley: those conferring photoperiod sensitivity (*Ppd*-*H1* and *Ppd*-*H2)*, requirement for vernalization (*Vrn*-*H1*, *Vrn*-*H2*, *Vrn*-*H3*) and earliness per se (*Eps*) genes (Cockram et al. 2007; Laurie et al. 1995; Snape et al. 2001). Among the contributing factors, *Eps* loci have been least investigated (Kamran et al. 2014). Unlike photoperiod and vernalization responses, *Eps* genes affect flowering time independently from environmental stimuli. Consequently, they may provide breeders with useful variation for fine-tuning flowering time (Laurie 1997). Several different *Eps* QTLs have been mapped to the pericentromeric region of chromosome 2H (Boyd et al. 2003; Cuesta-Marcos et al. 2008; Franckowiak and Konishi 2002; Horsley et al. 2006; Laurie et al. 1995; Moralejo et al. 2004; Pillen et al. 2003, 2004). This makes chromosome 2H an interesting target for further analyses.

Over the years, the simple diploid genetics of barley has been extensively explored in mutation research providing excellent resources for forward genetics (Lundqvist and Franckowiak 2003; Saisho and Takeda 2011). More than 1200 early-flowering mutant lines were isolated in barley between 1941 and 1988 at the Swedish Seed Association (later Svalöf AB), Sweden (Lundqvist 1992). Among them, 195 were tested in diallelic crosses, which revealed nine groups, *praematurum* (*mat)* -*a*, -*b*, -*c*, -*d*, -*e*, -*f*, -*g*, -*h* and -*i* (Lundqvist 1992), thus representing a valuable resource for deciphering the genetic control of flowering time in barley. The groups include photoperiod-insensitive (*mat*-*a* and *mat*-*e*) and photoperiod-sensitive mutants (*mat*-*b*, -*c*, -*d*, -*f*, -*g*, -*h* and -*i*) (Lundqvist 1992). In the present study, we exploited these comprehensive mutant resources for functional characterization of quantitatively inherited *Eps* genes. Similar to many other barley mutations, *mat*-*b.7* and *mat*-*c.19* have been introgressed into the spring barley cultivar Bowman by recurrent backcrosses (Druka et al. 2011). The resulting BW507 (*mat*-*b.7*) and BW508 (*mat*-*c.19*) lines were genotyped using high-throughput single-nucleotide polymorphism (SNP)-driven genotyping arrays, based on the Illumina GoldenGate oligonucleotide pool assay (Close et al. 2009), facilitating the assessment of their overlapping introgressions in the pericentromeric region of chromosome 2H (Druka et al. 2011). The potential co-localization of BW507 and BW508 introgressions on chromosome 2H gave a possibility to identify the underlying genes. The *mat*-*b* and *mat*-*c* mutant loci are particularly attractive due to the high number of available alleles (49 *mat*-*b*, 31 *mat*-*c*), increasing the chances to unambiguously connect *Mat*-*b* and *Mat*-*c* to particular gene models.

In the present study, we mapped the mutation in BW507 to a 31 Mbp interval on chromosome 2HL and identified the mutation as a deletion of the *Mat*-*c* gene. We conclude that both BW507 and BW508 are *HvCEN* deficient and none of them are derived from *mat*-*b.7*. Thus, the chromosomal location of *Mat*-*b* is unknown. This work complements the previous study by Comadran et al. (2012) by identifying the uncharacterized *mat*-*c.19* mutant and seven other *mat*-*c* mutants, and thus confirms the identity of *HvCEN* as *Mat*-*c*. In addition, we predict the effects of *mat*-*c* mutations at protein level by exploring the AtTFL1 protein 3D structure. We showed that most amino acid substitutions are accumulated at the potential ligand-binding site and the external loop underlining their importance for protein activity and overall structure stability.

## Materials and methods

### Plant material and growth condition

Barley plant material used in the present study is composed of two F_2_-mapping populations derived from the crosses BW507 × Bowman and BW507 × Barke, progeny of allelism crosses *mat*-*b.7* × *mat*-*c.19* and original *mat*-*b* (10 accessions) and *mat*-*c* (26 accessions) mutants, and corresponding parental lines BW507, BW508, Bowman, Barke, Bonus, Foma, Kristina and Frida. The parental lines are all spring barley cultivars. Plants were grown in greenhouse settings under long-day conditions (16 h light, 23 °C: 8 h dark, 20 °C) or in the field of southern Sweden (N 55°46′26.28″, E 13°13′26.47″). Plants were phenotyped for flowering time as days to emergence of the main spike awns at Zadoks 49 growth stage or days to the main spike heading at Zadoks 55 growth stage (Zadoks et al. 1974). To further confirm the phenotype, plants were scored for the number of kernels per main spike. Additional main tiller phenotyping concerning spike length, culm length, number and length of internodes was applied on the mature *mat*-*c* mutants, *mat*-*b.7*, BW507, BW508, F_1_ progeny of allelism crosses and the corresponding mother cultivars. Phenotypic data are presented as an average with standard deviations (± SD). Mutant seeds are available from the Nordic Genetic Resource Center (Alnarp, Sweden; http://www.nordgen.org).

### Allelism crosses

Allelism tests were carried out by crosses between *mat*-*c.19* and *mat*-*b.7*. Plants were emasculated and pollinated within 3 days. Nine F_1_ plants were obtained and phenotyped. Progenies of the *mat*-*c.19* × *mat*-*b.*7 cross were allowed to self-pollinate and flowering time frequency distribution was followed among F_2_ plants.

### DNA extraction and PCR analysis

Barley leaf fragments were collected from seedlings with three leaves and collected in 96-well plates. Genomic DNA extraction was carried out using modified protocol of the REDExtract-N-Amp Plant PCR Kit (Sigma-Aldrich, Germany) from either dry or fresh leaf material. The leaf samples were treated with 40 μl of extraction solution and incubated at 95 °C for 10 min followed by the addition of 40 μl of dilution buffer. DNA samples were stored at − 20 °C. Polymerase chain reactions (PCRs) were performed using REDExtract-N-Amp PCR ReadyMix (Sigma-Aldrich, Germany) with 10 μM of the respective PCR primers (Eurofins MWG Operon, Germany). The lists of specific PCR primers used to amplify SNP markers and candidate genes are provided in Supplemental Tables 1 and 2, respectively. PCR conditions were set as follows: initial denaturation at 94 °C for 5 min, 3 cycles of 94 °C for 30 s, 59 °C for 30 s, 72 °C for 45–90 s extension; 3 cycles of 94 °C for 30 s, 57 °C for 30 s, 72 °C for 45–90 s extension; 35 cycles of 94 °C for 30 s, 52 °C for 30 s, 72 °C for 45–90 s extension; final extension step of 72 °C for 5 min. Obtained PCR products were used as templates in DNA sequencing reactions for polymorphism determination. PCR products were purified with NucleoFast 96 ultrafiltration plates (Marcherey-Nagel GmbH & Co. KG, Germany), normalized and sent to the sequencing service offered by MWG-Biotech AG (Germany). The Freedom EVO 200 robot (Tecan Group, Switzerland) facilitated reaction mixing.

### Marker development and genotyping

The initial set of SNP markers used in genotyping of the mapping populations was established based on the previous analyses of the BW507 introgression line (Druka et al. 2011). For accessory marker information, barley genetic maps were explored (Close et al. 2009; Comadran et al. 2012). In total, 53 SNP markers (Supplemental Table 1) were tested for polymorphism determination. Among them, nine markers were not possible to amplify, other five markers did not show any polymorphism for involved parental lines and 12 markers were not linked to the BW507 mutant locus. Subsequently, 27 markers were used in the genetic mapping. SNP markers were either converted to cleaved amplified polymorphism sequence (CAPS) markers using NUBcutter v.2.0 webtool (http://tools.neb.com/NEBcutter2/) or assayed by DNA sequencing. Restriction digests were performed in a 20 µl reaction volume using 10 μl of PCR product and 1 unit of the restriction enzyme (New England Biolabs, USA). Reactions were incubated at 37 °C for a minimum duration of 1.5 h. DNA fragments were separated on 2% agarose gels for genotype scoring.

### Segregation analysis of *HvCEN*

Segregation analysis of *HvCEN* null allele was conducted in the BW507 × Bowman F_2_-mapping population consisting of 621 plants. Amplification of *HvCEN* was done with forward primer 5′-AGCCATCTCGTCTGTACACA-3′ and reverse primer 5′-GCAGATGTAGGTTGCACGTA-3′. The barley *Early*-*flowering 3* gene was used as an internal control and amplified as described elsewhere (Zakhrabekova et al. 2012) using respective forward 5′-GTCTGATTGGATTGGAAAACCTAG-3′ and reverse 5′-TGGGAAATTTTGCAGTTGG-3′ PCR primers.

### Statistical tests

Significant differences in the phenotypic traits analyzed between mutant and parental cultivars were calculated using a two sided Student’s *t* test. Phenotypic frequency distribution of kernel number per main spike and flowering time in F_2_ populations derived from the crosses BW507 × Bowman, BW507 × Barke and *mat*-*c.19* × *mat*-*b.7* were verified by *χ*^2^ test.

### Sequence homology searches

Barley DNA sequences with respective genetic and physical locations were extracted from Mascher et al. (2017, 2013) using the ViroBLAST interface tool (https://webblast.ipk-gatersleben.de/barley_ibsc/). Corresponding orthologous sequences of *Arabidopsis*, rice, Brachypodium and Sorghum were obtained from the Phytozome portal (https://phytozome.jgi.doe.gov/pz/portal.html). Alignments of the sequences were performed with the software package Jalview v2.7 (Waterhouse et al. 2009).

### Protein structure interpretations

Structural interpretation of *mat*-*c* mutations was carried out using the X-ray structure of the *Arabidopsis* TFL1, Protein Data Bank accession code 1WKO (Ahn et al. 2006) and rice FAC, Protein Data Bank accession code 3AXY (Taoka et al. 2011). Protein structures were visualized using the software Chimera (Pettersen et al. 2004).

### Accession numbers

The *mat*-*c.745* and *mat*-*c.1111* DNA sequences have obtained the GenBank accession numbers MN267905 and MN267906, respectively.

## Results

### Phenotype of *mat*-*b* and *mat*-*c* mutants

**Fig. 1:**
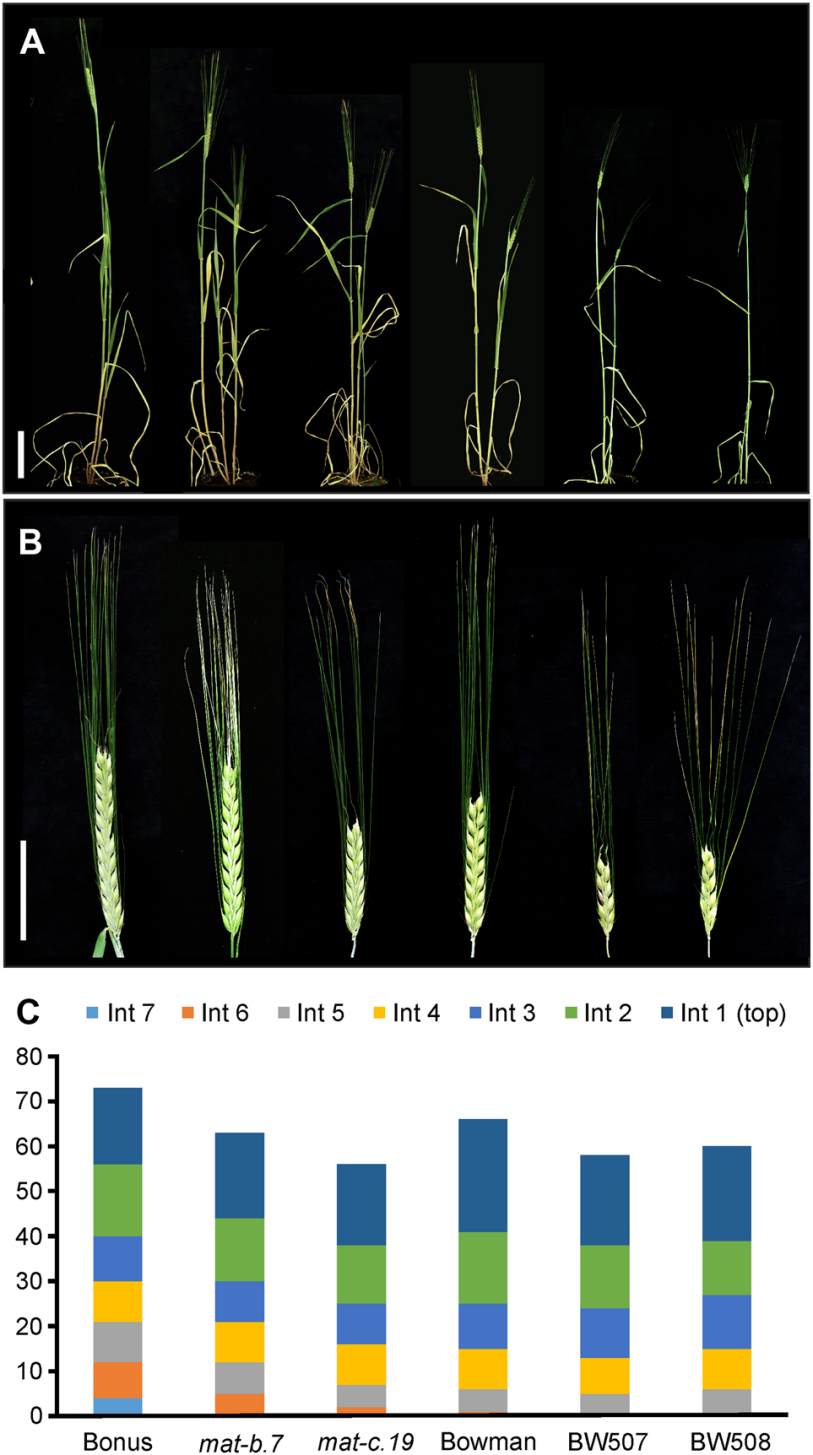
Phenotypic characterization of early-flowering *mat*-*b.7* and *mat*-*c.19* mutants in Bonus and Bowman genetic backgrounds. The mutations were originally induced in Bonus. Recurrent backcrosses generated the near-isogenic lines BW507 (supposed to be *mat*-*b.7*) and BW508 (*mat*-*c.19*). The label for the respective plant is shown in the lower part of the figure. **a** Overall plant architecture. Scale bar 10 cm. **b** Close-up of the main spike of each plant. Scale bars 10 cm. **c** Number and length (in cm) of culm internodes (Int)

Phenotypic effects of the *mat*-*b* and *mat*-*c* mutant loci were investigated using original *mat*-*b.7* and *mat*-*c.19* mutants induced in Bonus, and the respective Bowman introgression lines BW507 and BW508, grown in parallel with parental cultivars. Plants were cultivated in greenhouse settings (see “Materials and methods”) and phenotyped for flowering time as days to emergence of the main spike awns. Moreover, the penetrance of a potential pleiotropic effect on other traits of agronomic value was examined. The introgression lines BW507 and BW508 flowered 44 (± 0.71) and 45 (± 1.2) days after sowing, respectively, followed by Bowman at 49 (± 0.93) days. Mutant *mat*-*c.19* flowered at 53 (± 0.95) days after sowing, followed by *mat*-*b.7* at 54 (± 0.95) days and Bonus at 60 (± 2.1) days (Table 1). The early-flowering phenotype of analyzed mutant lines was accompanied by the following alterations in culm and spike architecture. The mutants were considerably shorter than the respective parental cultivar with maximum 90% of the wild-type culm length (Fig. 1a). The elongation of the basal-stem internodes was mainly decreased, while the upper-stem internodes remained less affected (Fig. 1c). In relation to culm length, the mutant plants developed approximately one internode less per culm than their parental cultivars. Furthermore, *mat*-*c.19* and Bowman introgression lines BW507 and BW508 developed short spikes with 60% of wild-type main spike length (Fig. 1b). This was associated with a decline in kernel number per main spike (Table 1). In contrast, the main spike architecture of *mat*-*b.7* resembled that of Bonus, with much less reduction in kernel number.

**Table 1:** Phenotypic characterization of *mat*-*b.7* and *mat*-*c.19* mutant lines and their parental cultivars

Name	Days to flower	Days earlier than wild-type	Culm length (cm)	Spike length (cm)	Kernel no.
Bonus	60.3 ± 2.1	–	71.4 ± 1.5	8.9 ± 0.47	22.2 ± 3.2
*mat*-*b.7*	54.6 ± 0.88***	5.8	63.9 ± 3.1***	8.6 ± 0.46*	18.0 ± 2.9**
*mat*-*c.19*	53.3 ± 0.95***	7.0	58.2 ± 4.8***	5.5 ± 0.50***	13.0 ± 1.2***
Bowman	48.9 ± 0.93	–	66.5 ± 4.7	7.6 ± 0.73	17.7 ± 1.2
BW507	44.2 ± 0.71***	4.6	57.3 ± 4.8***	4.1 ± 0.41***	6.0 ± 1.9***
BW508	44.6 ± 1.2***	4.3	59.7 ± 6.6*	4.5 ± 0.70***	6.7 ± 1.6***

Mutants *mat*-*b.7* and *mat*-*c.19* were induced in cultivar Bonus. BW507 and BW508 were reported to contain *mat*-*b.7* and *mat*-*c.19*, respectively, in a Bowman genetic background after recurrent backcrosses of the original mutants to Bowman (Druka et al. 2011). The phenotypic data include flowering time, culm length, spike length, and kernel number per main spike. The data are presented as an average with standard deviations (± SD) of nine replicates per accession grown under long-day conditions in greenhouse settings (20 °C). The flowering time was scored as days to awn emergence of the main spike. The significance was tested by a two-sided *t* test between the mutant and the respective parental cultivar. *p < 0.05, **p < 0.01, ***p < 0.001

### Allelism crosses between *mat*-*b* and *mat*-*c* mutants

On the basis of similar phenotypic characteristics and co-localization of the mutant donor introgressions, crosses between *mat*-*b.7 *× *mat*-*c.19* were performed to verify whether *mat*-*b* and *mat*-*c* complementation groups might be allelic. Plants were grown in the greenhouse settings and phenotyped for days to emergence of the main spike awns. The resulting F_1_ progenies, heterozygous for parental mutations, clearly resembled the phenotype of Bonus concerning flowering time, culm length, spike length and number of kernels (Table 2).

**Table 2:** Phenotypic characterization of F_1_ progenies derived from allelism crosses between *mat*-*b.7 *× *mat*-*c.19* and *mat*-*b.7 *× *mat*-*c.94* mutants in comparison to the respective mother cultivars

Name	Flowering time	Culm length (cm)	Spike length (cm)	Kernel no.
*mat*-*b.7*	59.3 ± 0.96***	80.4 ± 0.69***	9.7 ± 0.12***	26.3 ± 0.58***
*mat*-*c.19*	58.0 ± 1.0***	83.9 ± 0.86***	5.5 ± 0.14***	15.0 ± 0.82***
*mat*-*c.94*	53.4 ± 1.1***	74.4 ± 0.48***	5.7 ± 0.11***	15.0 ± 0.71***
F_1_, *mat.b.7* × *mat*-*c.19*	62.8 ± 1.3**	92.5 ± 5.1	10.1 ± 0.30**	28.3 ± 0.77***
F_1_, *mat.b.7* × *mat*-*c.94*	62.5 ± 1.2**	86.6 ± 4.5***	9.9 ± 0.49**	26.0 ± 0.1***
Bonus	64.5 ± 1.1	93.8 ± 1.3	10.6 ± 0.26	30.2 ± 0.96

The phenotypic data include flowering time, culm length, spike length, and kernel number per main spike. The data are presented as an average with standard deviation (± SD) of nine replicates per accession, grown under long-day conditions in greenhouse settings (15 °C). The flowering time was scored as days to awn emergence of the main spike. The significance was tested by a two-sided *t* test between the mutant and the mother cultivar Bonus. *p < 0.05, **p < 0.01, ***p < 0.001

**Fig. 2:**
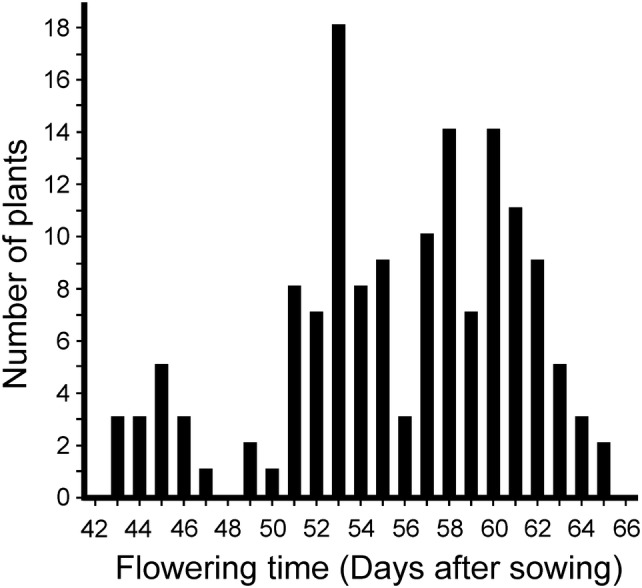
Flowering time frequency distribution among F_2_ progenies derived from a cross between *mat*-*b.7* and *mat*-*c.19*

To further confirm that *mat*-*b* and *mat*-*c* mutants are non-allelic, the progenies of the *mat*-*c.19 *× *mat*-*b.*7 cross were allowed to self-pollinate and flowering time frequency distribution was followed among 146 F_2_ plants (Fig. 2). The observed segregation in the population suggested a trimodal distribution (9:6:1 ratio, *p* = 0.12, *χ*^2^ = 4.26). In total, 15 F_2_ lines flowered drastically early at 45 (± 2.39) days after sowing, followed by a group of 53 F_2_ lines that flowered at 53 (± 1.94) days after sowing. The remaining 78 F_2_ lines flowered significantly later at 61 (± 1.92) days after sowing along with Bonus. The flowering time of the original *mat*-*b.7* and *mat*-*c.19* mutants overlapped with the F_2_ group flowering 53 days after sowing. The data suggest that the first F_2_ group corresponds to *mat*-*b.7 mat*-*c.19* double mutants, the second group corresponds to *mat*-*b.7* and *mat*-*c.19* single mutants and the third group corresponds to plants being heterozygous for the mutant alleles or homozygous for the wild-type *Mat*-*b* and *Mat*-*c* alleles.

### Molecular mapping of the BW507 early-flowering mutant locus

**Fig. 3:**
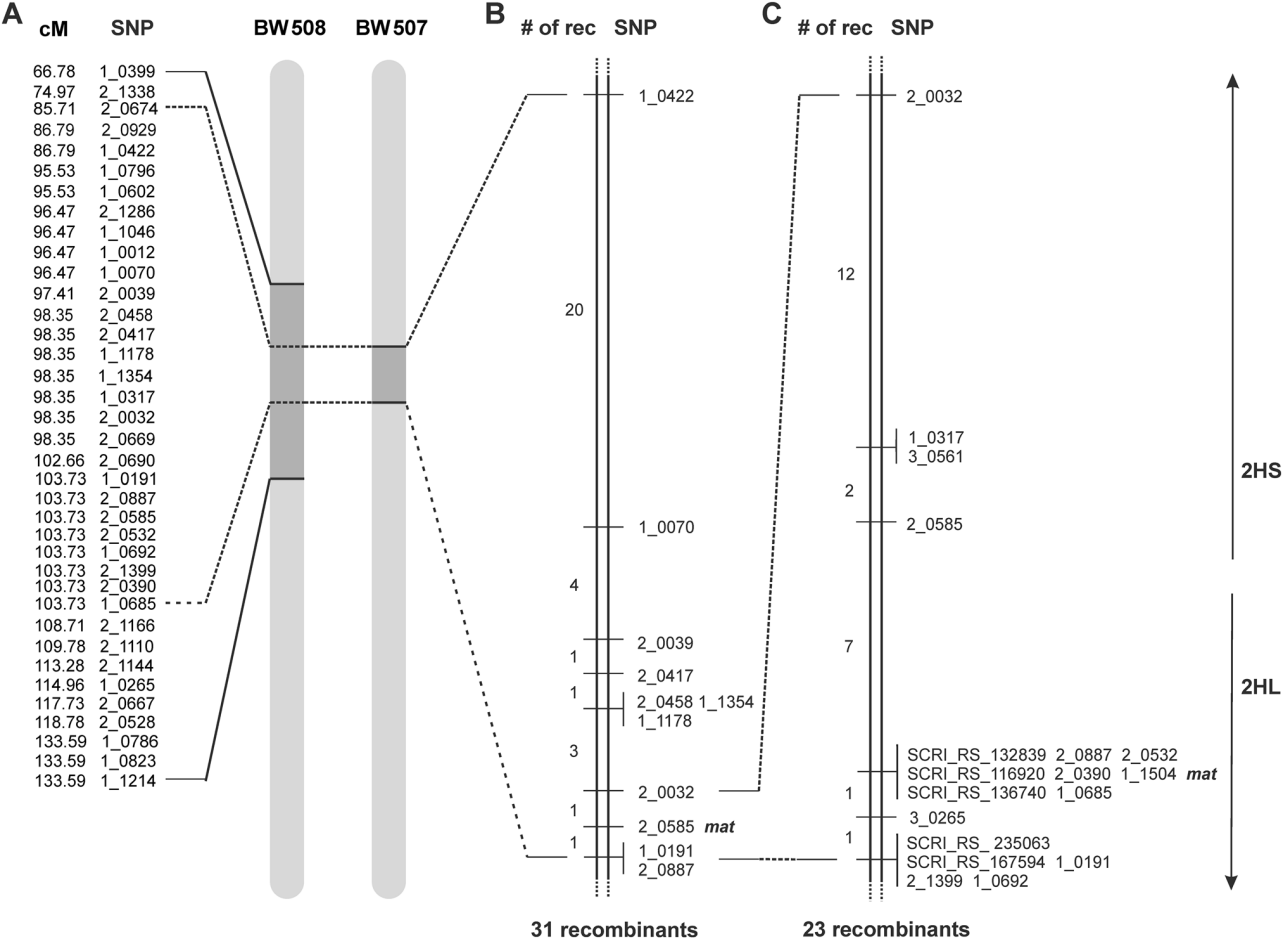
Molecular mapping of the mutation in BW507 that coincides with the BW508 introgression region. **a** The BW508 introgression on barley chromosome 2H covers 66.81 cM (37 SNP loci) flanked by BOPA markers 1_0399 (at 66.78 cM) and 1_1214 (at 133.59 cM). The BW508 introgression overlaps the BW507 introgression on 2H, defined by 25 SNP markers mapped within 18.02 cM and flanked by BOPA markers 2_0674 (85.71 cM) and 2_1399 (103.73 cM) (Druka et al. 2011). **b** Low-resolution mapping of the mutation in BW507. The mutation in BW507 was mapped to chromosome 2H using a BW507 × Bowman mapping population and 11 markers from the previously published map (Druka et al. 2011). The mutation was mapped between marker 2_0032 (98.35 cM) and a cluster of two markers 1_0191 and 2_0887 (103.73 cM). The position of mapped SNP markers follows the previously published map of the BW507 introgression (Druka et al. 2011). **c** High-resolution mapping of the mutation in BW507. The mutation in BW507 was fine mapped between markers 2_0585 and 3_0265. Arrows indicate which markers of the **c** panel reside on the short and the long arm of the chromosome 2H

Previous studies showed that the near-isogenic line BW507 carries the early-flowering mutant locus within one of the two introgressed segments, either on chromosome 2H defined by 25 SNP markers encompassing the genetic distance of 18.02 cM, or on chromosome 4H with 3 SNP markers mapped into a 6.45 cM interval (Druka et al. 2011). The BW507 introgression on chromosome 2H entirely overlaps with the single introgression of BW508 line (Fig. 3a) that carries the *mat*-*c.19* mutation within a 66.81 cM interval defined by 37 SNP markers (Druka et al. 2011).

**Fig. 4:**
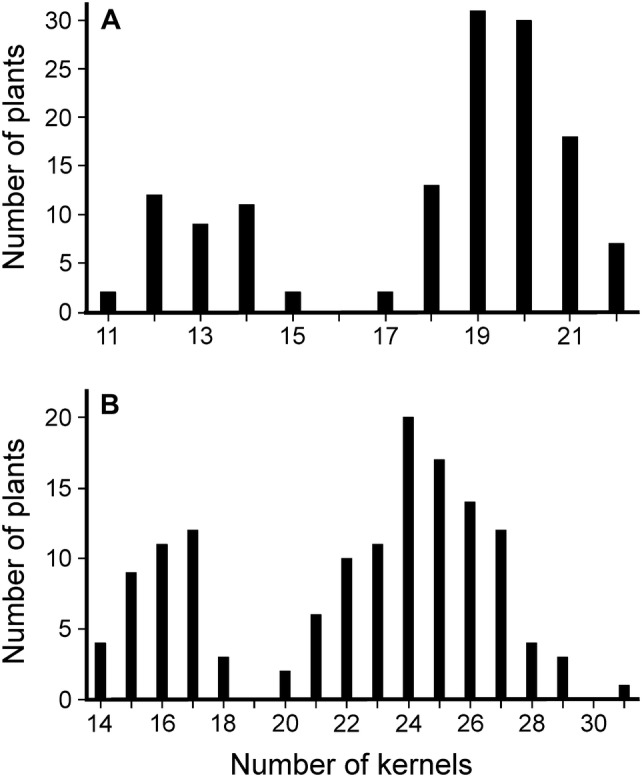
Phenotypic frequency distribution of kernel number per main culm in two F_2_ populations. **a** BW507 × Bowman, 137 lines. **b** BW507 × Barke, 139 lines

To establish a definitive chromosomal location of the BW507 mutant locus, a low-resolution bi-parental genetic mapping was performed on two F_2_-mapping populations derived from crosses of BW507 to the barley cultivars Bowman and Barke. Subsequently, 137 and 139 F_2_ lines were obtained. The mutant phenotype was initially scored based on heading day of the main spike. Despite the recessive character of the BW507 mutation and its distinctive early-flowering phenotype, the observed phenotypic frequency distribution for flowering time in the analyzed F_2_ populations did not correspond to that of a single-gene model inheritance. Phenotypic frequency distribution was presumably corrupted by the segregation of an additional early-flowering gene present in the Bowman genome as well as in BW507. Instead, kernel number per main spike was identified as an additional phenotypic character controlled by the BW507 mutant locus (Table 1). This enabled the inheritance of the BW507 phenotype to be followed as a Mendelian factor. The segregation ratio for kernel number in the BW507 × Bowman and BW507 × Barke populations (Fig. 4) was not considerably different from 1:3 (*p *= 0.69, *χ*^2^ = 0.73; *p *= 0.82, *χ*^2^ = 0.41). In the BW507 × Bowman population, 36 individuals showed significantly lower number of kernels (range 11–15, average ± SD 13.0 ± 1.1) and 101 yielded higher number of kernels (17–22, 19.5 ± 1.2). Correspondingly, in the BW507 × Barke population, 39 plants exhibited lower number of kernels (14–18, 16.0 ± 1.1) and 100 developed spikes with higher number of kernels (20–31, 25.5 ± 2.2). The average kernel number of both groups was significantly different from each other (BW507 × Bowman, *p *< 0.99, *t* test = 3.23E−04; BW507 × Barke, *p *< 0.99, *t* test = 5.17E−05).

Initial genotype analysis with SNP markers 2_1228 and 1_0319 flanking the BW507 introgression on chromosome 4HS (Druka et al. 2011) did not show any linkage with the mutant phenotype. We, therefore, excluded 4HS as a possible location for the mutation. Instead, the mutant phenotype was consistently linked with markers defining the BW507 introgression on 2H. The BW507 × Bowman mapping populations was screened with two markers flanking the BW507 introgression on 2H, 1_0422 and 2_0887 (Fig. 3a, b). This led to the identification of 31 recombinant lines, which were subsequently screened with additional nine markers that showed polymorphism between the original lines. The identified recombinants were self-pollinated and their phenotype was re-evaluated in the respective F_3_ progenies. We successfully narrowed down the location of the mutant loci to a region between marker 2_0032 and a cluster of two markers 1_0191 and 2_0887 in the pericentromeric region of chromosome 2H (Fig. 3b). According to the genetic distances used in Druka et al. (2011), we decreased the original 18.02 cM introgression to 5.38 cM. The population BW507 × Barke presented very low level of polymorphism and did not contribute to a higher resolution of the map.

To enhance genetic resolution around the BW507 mutant locus, high-density genetic mapping was performed using another 621 F_2_ plants from the BW507 × Bowman population. Moreover, F_2_ and F_3_ lines of BW507 × Bowman that remained heterozygous for the target interval were advanced to the next generation and subsequently used. In total, 1248 lines were genotyped with three flanking markers (2_0032, 2_0585 and 1_0191) and phenotyped for the number of kernels of the main spike. Twenty-three recombinant lines were obtained and screened with additional markers. Forty markers were initially selected based on previously published barley maps (Close et al. 2009; Comadran et al. 2012). Of these, 15 gave amplicons and showed polymorphism between involved parental lines. Subsequent genotyping allowed the BW507 mutant locus to be mapped between markers 2_0585 and 3_0265 on chromosome 2H (Fig. 3c). Marker 2_0585 is located at bp 494,259,016 on chromosome 2H and marker 3_0265 at bp 525,481,435 according the barley physical map (Mascher et al. 2017). The 31 Mbp region between the two markers contains 129 high-confidence genes (Supplemental Table 3) (Mascher et al. 2017). This should be compared with the original 18.02 cM introgression region of BW507 determined by Druka et al. (2011), which corresponds to 440 Mbp comprising 1581 high-confidence genes.

### Identification of *HvCEN* as a candidate gene underlying the BW507 mutant locus

The annotated function of genes predicted to be located in the target region was interpreted in context of known flowering time regulating genes. Four barley candidate genes HORVU2Hr1G037990, HORVU2Hr1G056040, HORVU2Hr1G063950 and HORVU2Hr1G072750 homologous to *Arabidopsis WITH NO LYSINE (K) KINASE 1* (*WNK1*, AT3G04910), *EARLY FLOWERING5* (*ELF5*, AT5G62640), *VERNALIZATION INDEPENDENT4* (*VIP4*, AT5G61150) and *TFL1* (AT5G03840), respectively, were identified. The candidate genes were sequenced from BW507, BW508, a set of *mat*-*b* mutants, *mat*-*c.19* and the mother cultivars Bowman, Bonus and Foma. Sequencing of *HvWNK1*, and *HvVIP4* genes did not reveal any polymorphism. However, polymorphism was found between Bowman and BW507 in *HvELF5*. Sequencing of *HvELF5* amplicons derived from the panel of recombinant plants from the BW507 × Bowman population mapped the single SNP to the vicinity but nine recombination events proximal to the delimited mutant locus. Additionally, the segregation analysis among F_2_ lines of BW507 × Bowman with a mutant phenotype did not agree with the genetic status at *HvELF5*. Amplification of the *AtTFL1* ortholog, *HvCEN*, did not give any amplicon in BW507 suggesting a putative deletion. However, all amplicons were obtained from the original *mat*-*b.7* mutant as well as all other tested *mat*-*b* mutants and mother cultivars. In addition, sequencing of these amplicons did not reveal any mutation and, therefore, *HvCEN* was excluded as a candidate of *Mat*-*b*.

**Fig. 5:**
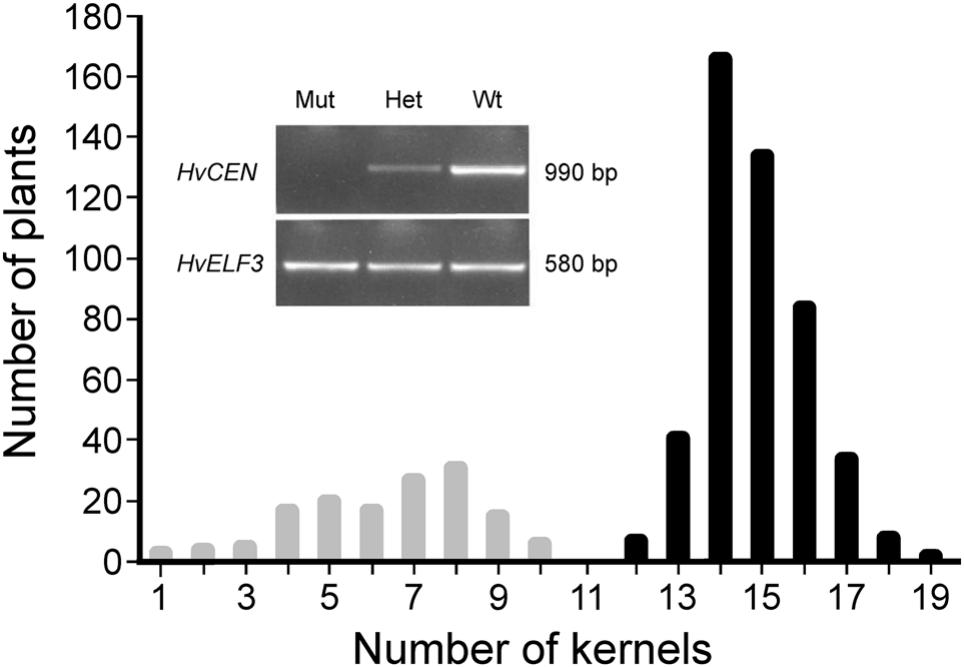
The variation in number of kernels in a BW507 × Bowman F_2_-mapping population consisting of 621 lines. The plants were phenotyped for number of kernels per main spike. 146 lines had less than 11 kernels and 475 lines had more than 11 kernels. All lines were genotyped for *HvCEN*. Gray bars represent lines being homozygous for the deletion of *HvCEN*. Black bars represent lines being heterozygous or homozygous for the wild-type allele. The insert illustrates the PCR-based genotyping procedure. DNA fragment of 990 bp was amplified from *HvCEN* and separated by agarose-gel electrophoresis. The mutation in BW507 represents a null allele of *HvCEN* allowing for dominant scoring. Correspondingly, F_2_ lines heterozygous (Het) or homozygous for the wild-type allele (Wt) gave an *HvCEN* amplicon of 990 bp. This was associated with higher kernel number in these lines. In contrast, F_2_ lines with the mutant allele of *HvCEN* did not give any amplicon and produced a lower number of kernels. In parallel, amplification for *HvELF3* was carried out as positive control

To evaluate the link between the deletion of *HvCEN* and the mutant phenotype of BW507, a segregation analysis was conducted among the 621 BW507 × Bowman F_2_ plants used for high-resolution mapping (Fig. 5). The 146 plants in which *HvCEN* was absent and 475 plants in which *HvCEN* was present (*p *= 0.822 and *χ*^2^ = 0.39 for 1:3 ratio) showed an exact co-segregation with the kernel number distribution in the F_2_-mapping population (Fig. 5). This observation strongly supported the identity of the *HvCEN* deletion as the mutation present in BW507. Therefore, contrary to the previous report (Druka et al. 2011), the BW507 introgression line is not *mat*-*b.7* derived. Interestingly, both BW508 and the original *mat*-*c.19* mutant, which previously failed to be identified as *HvCEN* deficient (Comadran et al. 2012), did not give *HvCEN* amplicons either. These findings strongly suggest that both near-isogenic lines BW507 and BW508 together with the original *mat*-*c.19* mutant carry a deletion of the genomic region where the *HvCEN* gene is located.

Comadran et al. (2012) identified 15 *mat*-*c* lines as *HvCEN* deficient. However, 16 other *mat*-*c* mutants remained genetically uncharacterized (Comadran et al. 2012). Sequencing of *HvCEN* from previously uncharacterized *mat*-*c* mutants led in the present study to the identification of a homozygous single-nucleotide polymorphism [c154a (numbering refers to HORVU2Hr1G072750)] resulting in a nonsynonymous amino acid substitution (Pro-52-Thr) in *mat*-*c.745*, a splice-site mutation originating from a g201a point mutation in *mat*-*c.1111* and entire gene deletion in *mat*-*c.16*, *mat*-*c.19*, *mat*-*c.122*, *mat*-*c.1107*, *mat*-*c.1108*, *mat*-*c.1096*. Sequencing of *HvCEN* from *mat*-*c.101*, *mat*-*c.758*, *mat*-*c.760*, *mat*-*c.865*, *mat*-*c.881*, *mat*-*c.910* and *mat*-*c.926* did not show any mutations. This suggested that the mutations are located outside the sequenced region, possibly in a regulatory element of *HvCEN* or that the accessions are wrongly annotated.

### Phenotypic characterization of *mat*-*c* mutants

The *Mat*-*c* locus is represented by an array of 31 recessive mutant alleles isolated in a wide range of cultivars after treatment with a whole spectrum of mutagens (Supplemental Table 4). For detailed phenotypic characterization, all *mat*-*c* mutants were grown under field conditions. Plants were phenotyped for flowering time, culm length, spike length and number of kernels of the main spike (Table 3). The combined genetic analyses by us and Comadran et al. (2012) revealed 24 *HvCEN* mutations that aligned with the early-flowering phenotype. The *mat*-*c* mutants flowered between 4 and 12 days earlier than corresponding mother cultivars. However, there was no link between expression of the earliness and the mutation type as both extremes were present among mutants with large deletion and those with a single point mutation. Similarly, no correlation could be seen between the phenotypic strength of the mutations and the location of the affected amino acid residue in the polypeptide sequence, i.e., whether the affected amino acid residue was located in the putative 14-3-3 interaction site, the potential ligand-binding site or the external loop (Table 3, Supplemental Figs. 1 and 2). Moreover, all mutations had pleiotropic effects on main culm and spike length. Mutants were considerably shorter and developed short spikes with lower number of kernels.

**Table 3:**
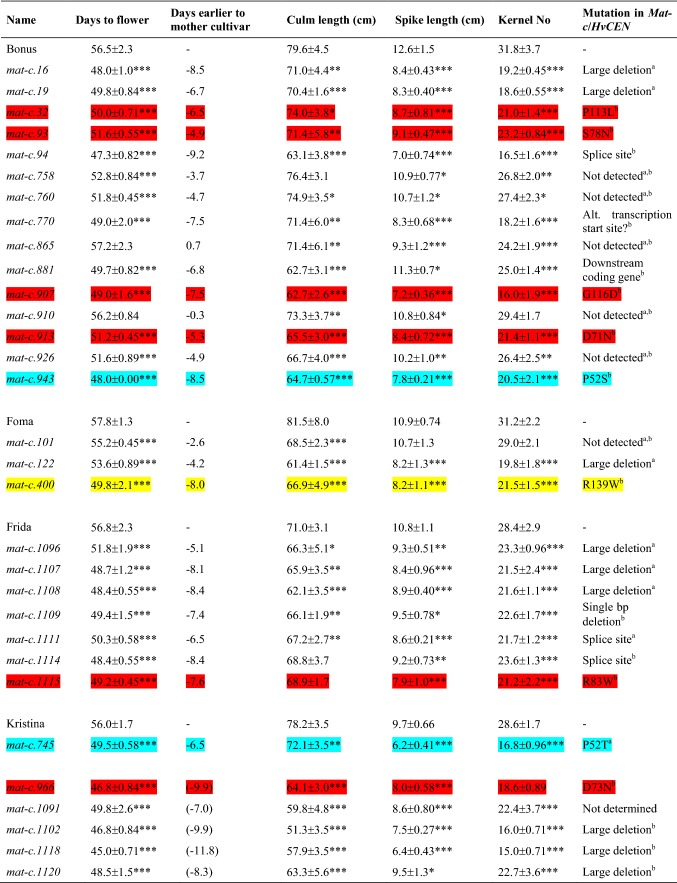
Phenotypic characterization of the 31 available *mat*-*c* mutants in comparison to the respective mother cultivars Bonus, Foma, Frida and Kristina

The mother cultivars of *mat*-*c.966* and -*c.1091* are Sv 79353 and Sv Ög 74233, respectively, which are no longer available. We did not have access to seeds of Semira, which is the mother cultivar of *mat*-*c.1102*, -*c.1118* and -*c.1120*. These five mutant lines were, therefore, compared to an average of Bonus, Foma, Frida and Kristina and their values are placed in brackets. The phenotypic data include flowering time, culm length, spike length and number of kernels per main spike. The earliness was accompanied by reduction in culm and spike length as well as a lower number of kernels per main spike. No mutations could be found in *Mat*-*c* of seven accessions and they did not display a pronounced reduction in culm length, spike length and number of kernels. The phenotypic data are presented as an average with standard deviation (± SD), of eight replicates per each accession. The flowering time was scored as days to awn emergence of the main spike. The significance was tested by a two-sided *t* test between the mutant and the respective parental cultivar. *p < 0.05, **p < 0.01, ***p < 0.001. The numbering of amino acid residues refers to barley *Mat*-*c* sequence HORVU2Hr1G072750. Mutations determined in this study or by (Comadran et al. 2012) are indicated by a or b, respectively. The color coding matches Supplemental Figs. 1 and 2. That is, a point mutation affecting an amino acid residue in the putative 14-3-3 interactive site is marked in blue, the potential ligand-binding site in red and the external loop in yellow

Sequencing of *HvCEN* from *mat*-*c.101*, *mat*-*c.758*, *mat*-*c.760*, *mat*-*c.865*, *mat*-*c.881*, *mat*-*c.910* and *mat*-*c.926* did not reveal any mutation. However, phenotypic analyses of these mutants revealed that they are missing typical *mat*-*c* mutant characteristics. In two cases, *mat*-*c.865* and *mat*-*c.910*, the lines flowered at the same time as their mother cultivar Bonus, which suggests that these accessions have been lost and are no longer available. The other five lines, *mat*-*c.101*, -*c.758*, -*c.760*, -*c.881* and -*c.926*, showed a medium to strong earliness, but in contrast to true *mat*-*c* mutants the length of spike and number of kernels per spike were often more similar to the mother cultivars (Table 3). It is likely that these lines carry a mutation in an early-flowering gene, but different from *HvCEN*.

### Effect of *mat*-*c* mutations on protein level

**Fig. 6:**
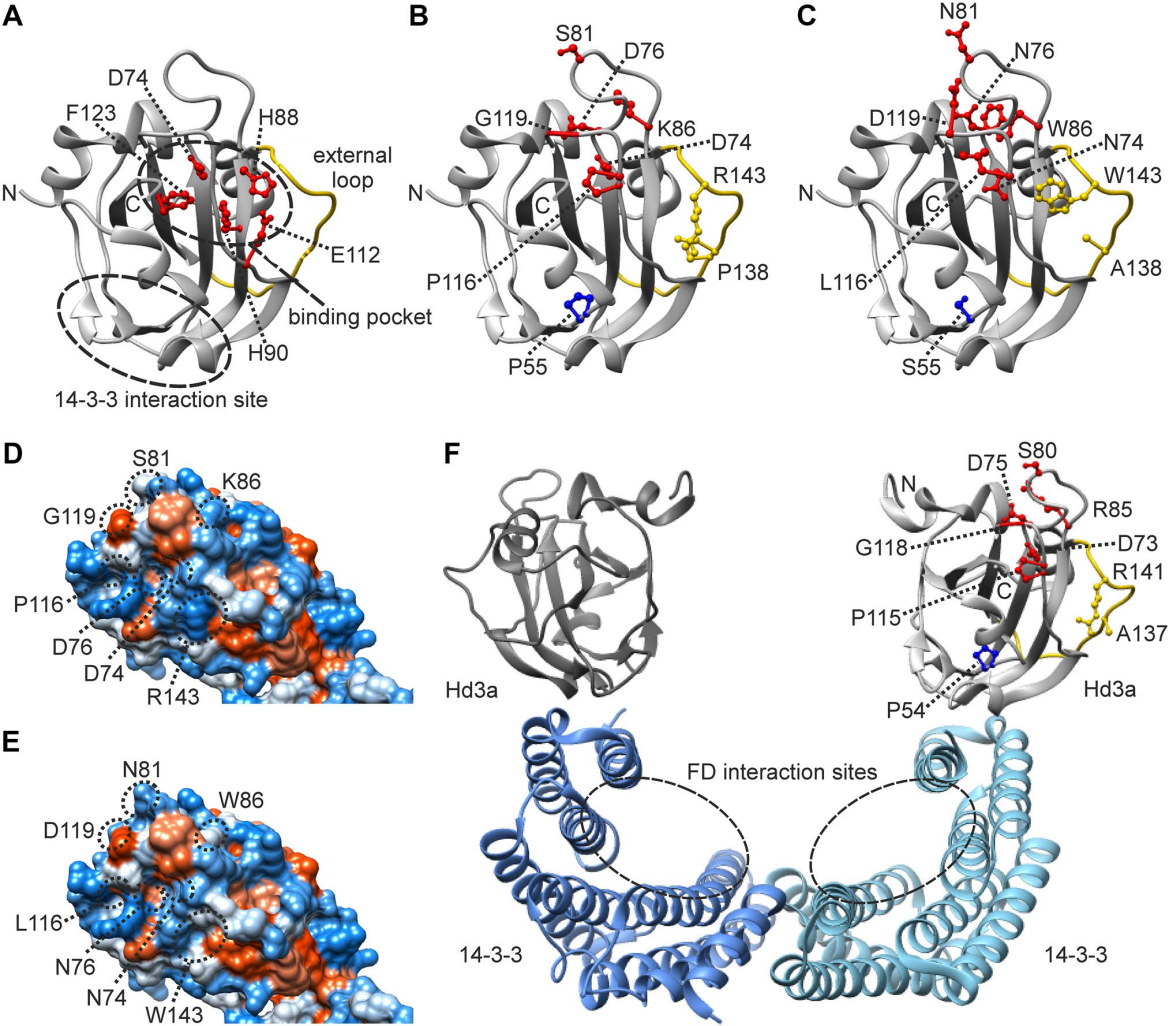
Structural implication of *HvCEN* point mutations. **a**–**e** X-ray structure of the Arabidopsis TFL1 (Protein Data Bank accession code 1WKO). The numbering of amino acid residues refers to the Arabidopsis TFL1 polypeptide sequence. **f** X-ray structure of the rice florigen activation complex (3AXY). The numbering of amino acid residues refers to the rice Hd3a polypeptide sequence. The alignment of AtTFL1, OsHd3a and HvCEN in Supplemental Fig. 2 provides the key for the translation of the residues between the three proteins. **a** Important domains and amino acid residues for the function of AtTFL1. Functional residues of the ligand-binding pocket are shown in red, the external loop is shown in yellow and the predicted 14-3-3 interaction site is marked with a circle. **b** The amino acid residues that are affected by mutations in *HvCEN*. **c** The amino acid residues resulting from the mutations in *HvCEN*. **d**, **e** Surface polarity changes due to identified substitutions. The two views **d**, **e** are shifted by 90° relative to (**a**–**c**). **f** The rice florigen activation complex (FAC) including Hd3a that interacts with the C-terminal domains of two 14-3-3 proteins. The putative OsFD interaction sites are indicated by circles. The ligand-binding pocket and external loop are exposed on the surface of the FAC structure and do not overlap with the 14-3-3 binding site. The amino acid residues affected by mutations in *HvCEN* are stated

The protein structure of AtTFL1 is very similar to that of AmCEN and other members of the PEBP family of proteins including *Arabidopsis* FT and rice Hd3a (Ahn et al. 2006; Banfield and Brady 2000; Hanzawa et al. 2005; Ho and Weigel 2014). Therefore, we can use the protein structure of AtTFL1 to map amino acid residues affected by different *mat*-*c* mutations (Supplemental Fig. 1). The AtTFL1 polypeptide sequence consists of 177 amino acid residues. The X-ray structure of AtTFL1 comprises residues 7-171 (Ahn et al. 2006). The structure is characterized by a central β-sheet flanked by a smaller β-sheet on one side and an α-helix on the other (Fig. 6a). Two regions of AtTFL1 have been found critical for inhibition of flowering. These are a potential ligand-binding pocket and an external loop also referred to as segment B (Ahn et al. 2006; Hanzawa et al. 2005; Ho and Weigel 2014). The binding pocket is defined by the residues Asp-74, His-88, His-90, Phe-123 and Glu-112, which have been previously suggested to play an important role in binding to phosphorylated interacting partners (Ahn et al. 2006; Ho and Weigel 2014). The external loop spans residues 128-145 located adjacent to the residues forming the ligand-binding site (Fig. 6a). The amino acid exchanges found in *mat*-*c.32* (Pro-113-Leu, Pro-116 in AtTFL1), *mat*-*c.93* (Ser-78-Asn, Ser-81 in AtTFL1), *mat*-*c.907* (Gly-116-Asp, Gly-119 in AtTFL1), *mat*-*c.913* (Asp-71-Asn, Asp-74 in AtTFL1), *mat*-*c.966* (Asp-73-Asn, Asp-76 in AtTFL1), and *mat*-*c.1115* (Arg-83-Trp, Lys-86 in AtTFL1) are located in the ligand-binding site (Fig. 6b, c). The mutation, *mat*-*c.400* (Arg-139-Trp, Arg-143 in AtTFL1), affects the external loop (Fig. 6b, c). The substitutions caused by the described mutations can be expected to have severe structural as well as direct functional effects on HvCEN. Changes in the surface polarity are also expected which would simply hamper potential protein interactions (Fig. 6d, e).

TFL1 has been reported to interact with FD in a complex, similar to FT (Hanano and Goto 2011). In addition, the tomato (*Solanum lycopersicum*) ortholog of TFL1, SP, and the rice ortholog of FT, Hd3a, have been shown to interact with 14-3-3 proteins (Okushita-Terakawa et al. 2018; Pnueli et al. 2001; Tsuji et al. 2009). In rice, it was suggested that 14-3-3 proteins mediate interaction between FD and Hd3a (Taoka et al. 2011). No direct interactions between FD and Hd3a could be detected by NMR titration assay, isothermal titration calorimetry, or a pull-down assay (Taoka et al. 2011). A crystal structure of the so-called florigen activation complex was reported (Taoka et al. 2011). The heterohexameric complex is composed of two Hd3a, two 14-3-3 proteins and two FD molecules (Fig. 6f). Each 14-3-3 protein has one Hd3a bound to the C-terminal region. The overall two-fold symmetric complex forms a thick and deep W-shaped structure. The phosphorylated C-terminus of FD binds to positively charged pockets formed within two corners of the inner base of the W-shape. We used the published florigen activation complex structure to locate amino acid residues affected by identified *mat*-*c* mutations with respect to potential protein interaction sites in the florigen activation complex (Fig. 6f). Pairwise protein sequence comparison of OsHd3a and Mat-c/HvCEN identified 101 identical amino acid residues (58%) representing notable sequence homology. Amino acid residue Pro-52 (Pro-54 in OsHd3a, Pro-55 in AtTFL1), affected by *mat*-*c.745* (Pro-52-Thr) and *mat*-*c.943* (Pro-52-Ser) is located in the potential 14-3-3 interaction site. Therefore, it is highly probable that the *mat*-*c.745* and *mat*-*c.943* mutations cause changes of amino acid residues that abolish potential interaction between Mat-c/HvCEN and 14-3-3.

## Discussion

An earlier study of barley near-isogenic lines suggested that the *Mat*-*b* and *Mat*-*c* loci, represented by near-isogenic lines BW507 and BW508, respectively, are closely located on chromosome 2H (Druka et al. 2011). In the present study, we described a high-resolution mapping of BW507, which was reported to carry the *mat*-*b.7* mutation in an 18.02 cM introgression interval (Druka et al. 2011). We assigned the mutation in BW507 to a 31 Mbp interval on chromosome 2HL (Fig. 3) from a mapping population consisting of more than 1000 lines. The interval coincides with the introgression region of BW508 carrying *mat*-*c.19* (Druka et al. 2011). Allelism tests with original *mat*-*b* and *mat*-*c* mutants proved that *Mat*-*b* and *Mat*-*c* are not allelic. Moreover, the two genes seem to work independently of each other since the double mutant flowered earlier than each of the individual mutants (Fig. 2). The observation indicates that the *Mat*-*b* and *Mat*-*c* gene products belong to different flowering pathways. Screening the functional annotations of genes presumably located in the target interval enabled us to identify the mutation in BW507 as a deletion of *HvCEN*, which is also present in BW508 corresponding to *mat*-*c.19,* but not present in the original *mat*-*b.7* mutant. Thus, a chromosomal location of *Mat*-*b* has still not been assigned. The present study and the study of Comadran et al. (2012) identify barley *Mat*-*c*/*HvCEN* as an ortholog of *Arabidopsis TERMINAL FLOWER1* (*AtTFL1*) and *A. majus CENTRORADIALIS* (*AmCEN*). When combined, the two studies identify severe mutations in 24 of the 31 *mat*-*c* mutant accessions. The remaining accessions where no mutations could be identified appear to have lost the early-flowering phenotype or do have an early-flowering phenotype that is not connected to the *Mat*-*c* locus. We have previously noted that some accessions of historic mutants in the *Mat*-*a*, *Clo*-*f2*, *Ari*-*o*, *Ert*-*m* and *Brh2*/*Ari*-*l* loci, handled by generation of breeders and researchers, have been mixed or lost (Braumann et al. 2018; Dockter et al. 2014; Mueller et al. 2012; Zakhrabekova et al. 2012, 2015).

The barley mutants belonging to the nine complementation groups, *mat*-*a*, -*b*, -*c*, -*d*, -*e*, -*f*, -*g*, -*h* and -*i* (Lundqvist 1992), show a variation of early flowering from moderate to drastic earliness. Mutants in the *Mat*-*c* locus belong to the drastic early group, still with some variation within the group (Table 3). Another drastic mutant group is *mat*-*a* (Zakhrabekova et al. 2012). The mutant *mat*-*a.8*, under the name Mari, was one of the first induced barley mutants to be released as a commercial cultivar (in 1961) (Dormling et al. 1966). In addition to an early-flowering phenotype, Mari barley also featured a ridged plant stature, which provided increased lodging resistance. Mutations in the *Mat*-*c* locus also caused pleiotropic effects; notably a slight reduction in culm length as well as a pronounced decrease in length of spike and number of kernels per spike. Thus, *mat*-*c* mutant alleles have negative effects on crop yield and are less attractive for plant breeding. Still, the characterized *mat*-*c* mutations are of scientific importance since they provide unique information about members of the PEBP family of proteins such as TFL1 and FT, which are key proteins regulating the transition from vegetative to reproductive growth of plants.

Despite the opposing functions, TFL1 and FT proteins are structurally highly similar (Hanzawa et al. 2005; Ho and Weigel 2014). TFL1 is a negative regulator of flowering. Therefore, the barley *mat*-*c* mutants show an early-flowering phenotype. In nine mutants, we found large deletions of the entire gene, whereas five mutants could be expected to have truncated proteins because of mutations in for example exon–intron splice sites. Nine mutants caused substitutions of single amino acid residues. These were analyzed in combination with available X-ray structures of TFL1 and FT proteins. In sequence alignments, the barley Mat-c/HvCEN polypeptide shows more than 50% identical amino acid residues to AtTFL1, AmCEN, AtFT, OsHd3a and other members of the PEBP-protein family. In comparison, the cytochrome *c* family, which is broadly acknowledged as a highly conserved evolutionary family with very similar protein structures, often shows invariance of about 40% of their residues (Banfield and Brady 2000). We used the protein structure of AtTFL1 (Ahn et al. 2006) to map amino acid residues affected by different *mat*-*c* mutations. This way of highlighting amino acid residues by connecting genotype and phenotype is especially important to gain knowledge about proteins involved in processes which are hard to measure and monitor in vitro compared to for example enzymes which can be functionally studied in enzyme assays. In mutants *mat*-*c.32*, *mat*-*c.93*, *mat*-*c.907*, *mat*-*c.913*, *mat*-*c.966* and *mat*-*c.1115*, six different amino acid residues in the potential ligand-binding pocket are affected (Fig. 6b, c). The exchange Gly-to-Asp in *mat*-*c.907* (Gly-119 and Gly-116 in AtTFL1 and Mat-c/HvCEN, respectively) is located in the junction between a loop and a β-strand and represents a drastic amino acid modification. The mutation in *mat*-*c.32* is also affecting the same loop (Pro-116 in AtTFL1, Pro-113-Leu in Mat-c/HvCEN). Although the polarity is conserved in a Pro-to-Leu substitution, the mutation is severe since the mutant *mat*-*c.32* has a similar loss-of-function phenotype compared to other *mat*-*c* mutants in terms of flowering and number of kernels. The charged residues Asp-74, Asp-76 and Lys-86 in the AtTFL1 ligand-binding pocket are neutralized in *mat*-*c.913* (Asp-71-Asn), *mat*-*c.966* (Asp-73-Asn) and *mat*-*c.1115* (Arg-83-Trp), respectively. The latter substitution to a tryptophan residue can additionally cause a structural change. The function of residue Ser-81, affected by *mat*-*c.93* (Ser-78-Asn), is less clear since it is located at the surface of the ligand-binding pocket. Possibly, Ser-81 is involved in interactions with another protein.

The external loop, also named segment B (Ahn et al. 2006; Hanzawa et al. 2005; Ho and Weigel 2014), is affected only by mutant *mat*-*c.400* in which Arg-143 (Arg-139 in Mat-c/HvCEN) has been changed to Trp. It is worth noticing that Pro-138 is also located in the external loop, five residues away from Arg-143. Resequencing of *Mat*-*c*/*HvCEN* from 216 spring and 207 winter barley accessions revealed polymorphism affecting Pro-138 (Pro-135 in Mat-c/HvCEN) (Comadran et al. 2012). All winter accessions, included proline in position 138, whereas alanine was specific to accessions with spring growth habit. Changes in the external loop were previously shown to be largely sufficient to convert TFL1 into an FT-like activator of flowering (Ahn et al. 2006; Hanzawa et al. 2005). It should be noted that barley, rice and *Arabidopsis* FT polypeptide sequences, contain alanine in the positions corresponding to Pro-135 in Mat-c/HvCEN (Ala-135 in HvFT1, Ala-137 in OsHd3a, Ala-135 in AtFT). The substitution of a proline to an alanine may destabilize the structure or open the structure to potentially interacting macromolecules. Two functionally diverged paralogs of *FT* have been identified in poplar, *FT1* and *FT2,* and were suggested to originate from a whole-genome duplication (Hsu et al. 2011). Physiological and genetic studies revealed that *FT1* determined reproductive onset in response to winter temperatures, whereas *FT2* promoted vegetative growth in response to warm temperatures and long days in the growing season (Hsu et al. 2011). The diverged functions of two paralogous proteins were attributed to changes of 16 amino acid residues. These include alanine in FT1 and proline in FT2 at position 136 in the poplar proteins. Pro-136 makes the external loop of FT2 more hydrophilic potentially affecting protein–protein interaction.

Our analyses of barley *mat*-*c* mutants were also associated with interaction sites in the suggested florigen activation complex (Taoka et al. 2011), which would specifically concern interactions between Mat-c/HvCEN and 14-3-3. The ligand-binding pocket and the external loop are exposed on the surface of the florigen activation complex and do not overlap with the 14-3-3 interaction site. Therefore, it is rather unlikely that mutations affecting amino acid residues in the ligand-binding pocket or the external loop would disturb potential interactions between Mat-c/HvCEN and 14-3-3. Instead, the two mutations *mat*-*c.745* (Pro-52-Thr) and *mat*-*c.943* (Pro-52-Ser) both affecting amino acid residue Pro-52 (Pro-54 in OsHd3a, Pro-55 in AtTFL1) located directly in the interaction site of Mat-c/HvCEN and 14-3-3 will most likely hamper their interaction (Fig. 6f). The large distance between the ligand-binding pocket and the external loop in relation to the 14-3-3 binding site suggests that other proteins may further interact with the florigen activation complex. The members of the TCP family of transcription factors were anticipated as potential candidates for mediating antagonistic functions of *Arabidopsis* TFL1 and FT (Cubas et al. 1999; Ho and Weigel 2014).

In summary, our study and the study by Comadran et al. (2012) reveal a rich repertoire of mutations in a key gene regulating early flowering. We further explore available protein structures to provide a reasonable explanation to the negative impact of changed amino acid residues resulting from identified nonsynonymous mutations. The identified *mat*-*c* mutant alleles have a negative effect on kernel number and length of spike, which suggest that *Mat*-*c*/*CEN*/*TFL1* is less suitable for barley breeding.

### Author contribution statement

IM and MH designed the experiments. IM, MT, SZ, CD, MH performed the research. IM and MH analyzed data and wrote the manuscript.

## Electronic supplementary material

Below is the link to the electronic supplementary material.

Supplementary material 1 (DOCX 1483 kb)
