# Supplementary material for: Analysis of early-flowering genes at barley chromosome 2H expands the repertoire of mutant alleles at the Mat-c locus
Source: Plant Cell Rep. 2019 Sep 20;39(1):47–61. doi: 10.1007/s00299-019-02472-4 (PMC6960220; doi:10.1007/s00299-019-02472-4)
Supplement: Supplementary file 1 — Supplementary material 1 (DOCX 1483 kb) [file 299_2019_2472_MOESM1_ESM.docx]

**Supplemental figures 1 and 2, and Supplemental Tables 1, 2 and 3, of the Plant Cell Reports publication:**

Analysis of early flowering genes at barley chromosome 2H expands the repertoire of mutant alleles at the *Mat-c* locus encoding a CENTRORADIALIS protein

**Authors:**

Izabela Matyszczak, Marta Tominska, Shakhira Zakhrabekova, Christoph Dockter and Mats Hansson

**Corresponding author:**

Mats Hansson, Lund University, Department of Biology, Sölvegatan 35, SE-22362 Lund, Sweden, mats.hansson@biol.lu.se, +46462224980, +46462224117


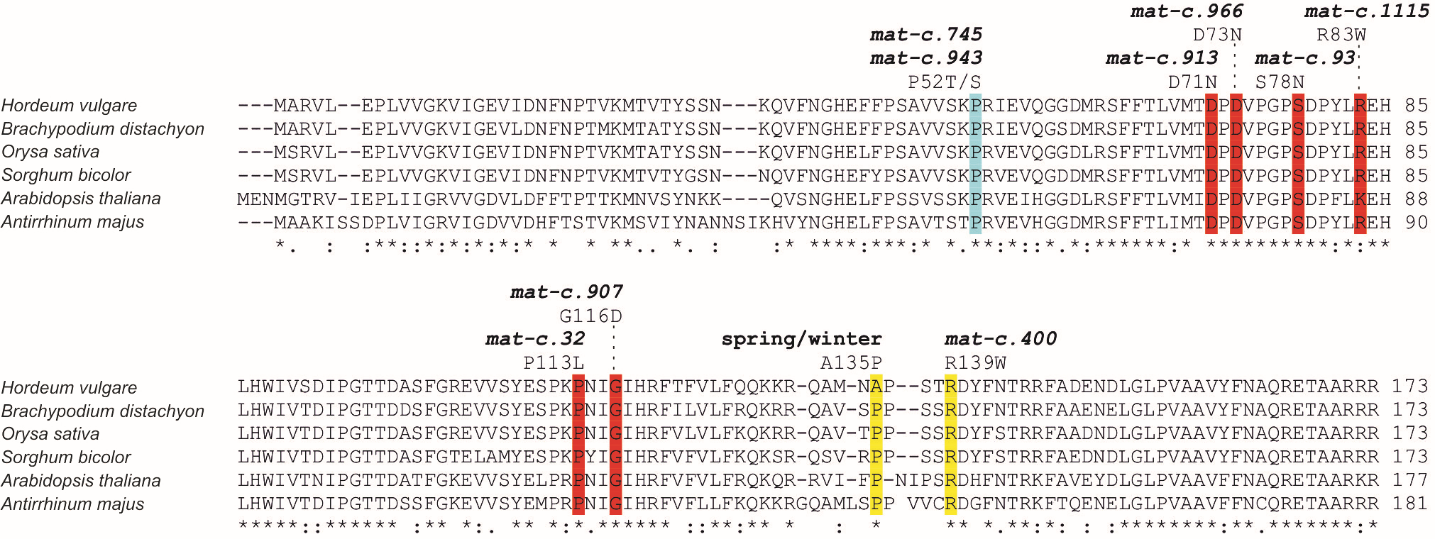


**Supplemental Fig. 1.** Alignment of polypeptides deduced from *HvCEN* and *TFL1/CEN*-like genes from different plant species. Identical amino-acid residues are marked with asterisks. Conserved substitutions are marked with colons. Semiconserved substitutions are marked with dots. The overall alignment shows 58% identical amino-acid residues. Colored amino-acid residues are affected by the identified point mutations in *mat-c* mutants causing the substitutions of amino-acid residues. All amino-acid exchanges are located at conserved positions of CEN, i.e. within the putative 14-3-3 interaction site (shown in blue), the potential ligand-binding site (red) and the external loop (yellow). The NCBI GenInfo Identifier number of each protein sequence in the alignment is: *Hordeum vulgare* GI:410442693, *Brachypodium distachyon* GI:357163323, *Orysa sativa* GI:115458266, *Sorghum bicolor* GI:242075692, *Arabidopsis thaliana* GI:15237535 and *Antirrhinum majus* GI:12195101.


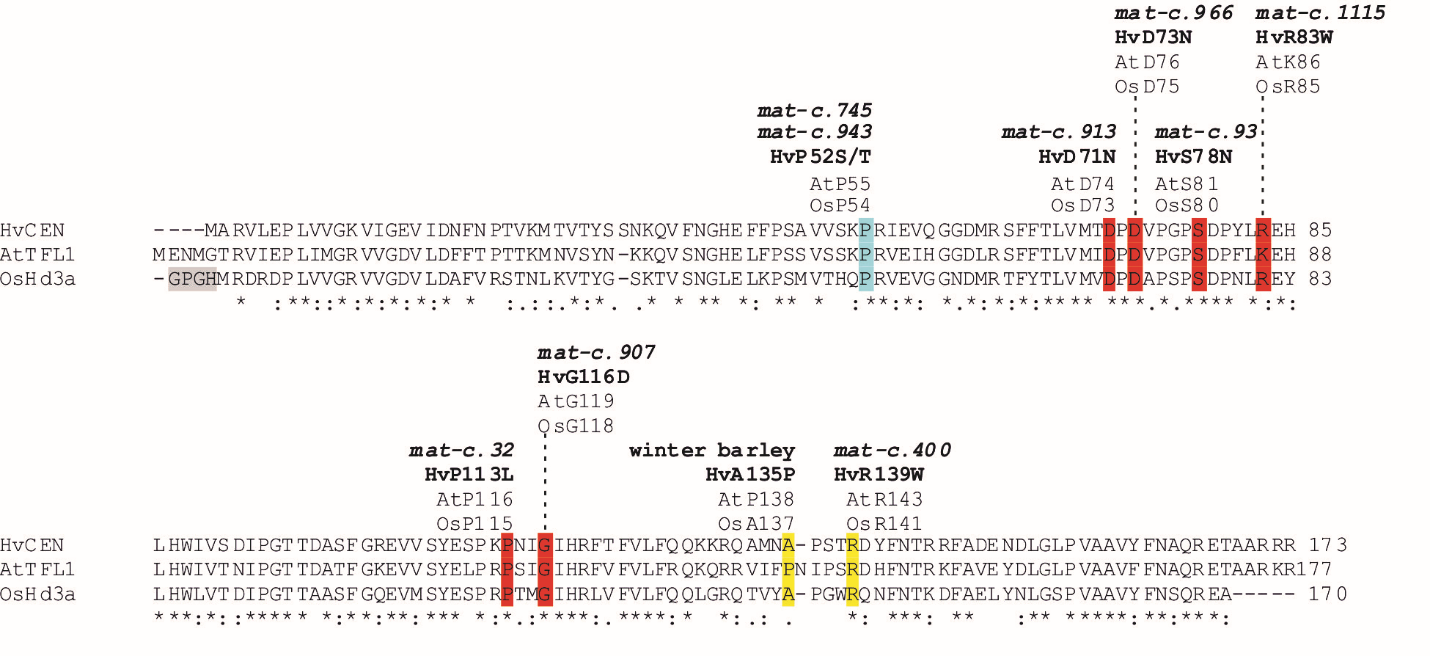


**Supplemental Fig. 2.** Alignment of AtTFL1, OsHd3a and HvCEN. The 3D structures of AtTFL1 (Protein Data Bank accession 1WKO) and OsHd3a (3AXY) are shown in Fig. 6. Indicated positions correspond to the amino-acid exchanges that have been identified in barley *mat-c* mutants. Their localization in AtTFL1 and OsHd3a protein sequences are shown. Mutations occurring within the putative 14-3-3 interaction site are shown in blue, the potential ligand-binding site in red and the external loop in yellow. OsHd3a residues (GPGH, marked in gray) have been artificially introduced during the crystallography procedure.

**Supplemental Table 1.** List of SNP markers tested for genetic mapping of F_2_ mapping populations BW507 × Bowman, BW507 × Barke and BW507 × Morex.

| **SNP** | **Source** | **Chromosome** | **Forward Primer (5'-3')** | **Reverse Primer (5'-3')** | **Polymorphism** | **Detection** |
| --- | --- | --- | --- | --- | --- | --- |
| 2_1228 | Druka et al., 2011 | 4H | CTGCATAACAACATCTGGTG | TCTGGTGTTGGTGTTATTGG | A/G | CAPS |
| 1_0319 | Druka et al., 2011 | 4H | CGTACAGTTACCCCAAGGAC | TCGAGTATATCCATTTCCGG | A/G | CAPS |
| 1_0297** | Close et al., 2009 | 2H | ATCGTCGATGACATGAGGAA | TGCACACCTTCAAATACCGT | - | - |
| 1_0325* | Close et al., 2009 | 2H | GGTTTCAGTTTCATCCAAAA | GGATCACCATAATTACAAGG | G/A | CAPS |
| 1_0436 | Close et al., 2009 | 2H | AAAACACGAGCTCCACAACT | TATGTGTTGTTTCAAAAGGG | No | - |
| 1_0638** | Close et al., 2009 | 2H | TAAGCTAAGCACATCTGCCA | TTATTAACCGGAAAGCTTGG | - | - |
| 1_0750 | Close et al., 2009 | 2H | AGGAGAAATGGAAGCAGCTA | CGGTATTTTTAGAAGCCTGG | No | - |
| 1_0997* | Close et al., 2009 | 2H | GAAAGCGACAGACATCCTAC | AGCGTAGAAGAAAGAAACCG | G/A | Sequencing |
| 1_1015* | Close et al., 2009 | 2H | GCTGATATTATCAAGGTGGG | AGCTGTGAGCTGAGATTGAT | G/A | Sequencing |
| 1_1206 | Close et al., 2009 | 2H | ATTCGCCACAGTTCATTTTT | GAATTTGATCCTTGCTGTCC | No | - |
| 1_1302** | Close et al., 2009 | 2H | GAGACAGGTAAACCAACCTC | ACAAATGCTAGTCCTTGGAG | - | - |
| 1_1384 | Close et al., 2009 | 2H | GGCGAACCTGGTCTACTTCT | CTTCTTGGAGACCTCGGATT | No | - |
| 1_1400* | Close et al., 2009 | 2H | GAGCGTGTGCTTAAACAGAT | CAATAACTTTTGCCCTTCAA | T/C | CAPS |
| 1_1504 | Close et al., 2009 | 2H | CCTTCTCAACCTTGCTCTTC | GGAGATTGTGGAGTCTGTGA | C/T | Sequencing |
| 1_1522* | Close et al., 2009 | 2H | CTTTCTCATTCCCCAGGACT | ACACGCACAACCAGATAAAC | T/C | Sequencing |
| 2_0387** | Close et al., 2009 | 2H | GATCTCTCCCTCGGCTCCAC | GAAACCGCGTCCTCCCTTAT | - | - |
| 2_0690 | Close et al., 2009 | 2H | GCTTGGTCAACAGTCTGTGT | AGGTCAATTGGCATAGTTGT | No | - |
| 2_0891* | Close et al., 2009 | 2H | GTGAGCCATTGACGTCTTAC | AGTCCAGTGCAACCTTTTCC | G/A | CAPS |
| 2_1096** | Close et al., 2009 | 2H | TCTTCTTCTTCTGATCGATG | TTTGGTTTATAGAAGATCCG | - | - |
| 2_1388* | Close et al., 2009 | 2H | CATGAGAGGTCGAGAGGTAG | AGTACATGTTGACCCATGAA | A/C | CAPS |
| 3_0338** | Close et al., 2009 | 2H | GGGGACTACTCTGCATTTCA | AGAGGTCAGAATGGTTTCAT | - | - |
| 3_0561 | Close et al., 2009 | 2H | CTTACGACCAGCCTAAAAGG | AAATTACGGGTGTGTTTCCC | A/T | Sequencing |
| 3_0265 | Close et al., 2009 | 2H | GTTGTTGCGCCTGTAGCCTT | TGCACCGAGAACATCATCTG | G/A | Sequencing |
| SCRI_RS_116920 | Comadran et al., 2012 | 2H | GGGTCTTATTGGGGAAGAAA | CCAGTCAGAAAATTGGATGA | G/A | Sequencing |
| SCRI_RS_132839 | Comadran et al., 2012 | 2H | CTGGACCTCAAGGACCCGTT | ATCTCCTGTGCAATGGGGAA | G/A | Sequencing |
| SCRI_RS_136740 | Comadran et al., 2012 | 2H | CAGGGTAAGCACTTATGGAG | TCATCGTATTCGGTATTTCC | C/A | Sequencing |

*Polymorphic markers localized outside the target interval therefore not analyzed in the mapping populations

** Unsuccessful amplification

**Supplemental Table 1.** Continued

| **SNP** | **Source** | **Chromosome** | **Forward Primer (5'-3')** | | **Reverse Primer (5'-3')** | | **Polimorphism** | | **Detection** |  |
| --- | --- | --- | --- | --- | --- | --- | --- | --- | --- | --- |
| SCRI_RS_167594 | Comadran et al., 2012 | 2H | TTTATCCTCATCACTTGCCA | AAGAAAAAACTTGCCTTGCC | | G/A | | Sequencing | | |
| SCRI_RS_235063 | Comadran et al., 2012 | 2H | CTTCTCTTCGGCAAGCTATA | AGGAGGAAAGGGAATTGAAG | | G/A | | Sequencing | | |
| 2_0674** | Druka et al., 2011 | 2H | CACCAAGGCCAGACACAC | GGTCCTGGGAGGGTTCAT | | - | | - | | |
| 2_0929** | Druka et al., 2011 | 2H | ACCGTCACCTTCGAGTTCG | ACAAGCAGATGCCAAGCG | | - | | - | | |
| 1_0012* | Druka et al., 2011 | 2H | GCTTTACTGACACATCCCTT | | GTCTTGGAACATCAAAACTC | | A/G | | Sequencing |  |
| 1_0070 | Druka et al., 2011 | 2H | GTGCTTGATCTCCGCCAG | | GGCAAGGTGGAGCTGGTG | | T/C | | CAPS |  |
| 1_0191 | Druka et al., 2011 | 2H | CCCTTATTGTAGAAATTACAGAGTCA | | GCTCTGCAATACGTCCAAGAA | | A/C | | CAPS |  |
| 1_0317 | Druka et al., 2011 | 2H | AAAGAAAAACAGCCTCCTCA | | CAATACCACCAAGCAAGTGG | | A/C | | Sequencing |  |
| 1_0422 | Druka et al., 2011 | 2H | TGATGAGGTCAACAATGCG | | GGCTGACAAAAATCTTCCG | | T/G | | CAPS |  |
| 1_0602* | Druka et al., 2011 | 2H | GCGGAACCCTTCAACAAGAC | | GAGGTGCTCCAGAAGTGGGT | | A/C | | Sequencing |  |
| 1_0685 | Druka et al., 2011 | 2H | AGCTACCCATGAAGCAATTACA | | TCGTTCACTTCCTGGGGAT | | T/C | | CAPS |  |
| 1_0692 | Druka et al., 2011 | 2H | TAACCTGCCAAGTTTTCAGG | | TTCTCAACAGATCTTTGGGA | | T/C | | Sequencing |  |
| 1_0796* | Druka et al., 2011 | 2H | ACTCTCCCTGATGGGTCGAG | | TGCATGGCTATGTAGTAGCG | | T/C | | Sequencing |  |
| 1_1046* | Druka et al., 2011 | 2H | CAGCGACTGTTTCAGAAGAG | | GATCCCAGTACAACAACCAG | | A/G | | Sequencing |  |
| 1_1178 | Druka et al., 2011 | 2H | CATGGTCAGCTAGGTAGGTC | | AGCAACTTCATCAGTTGGAT | | T/C | | Sequencing |  |
| 1_1354 | Druka et al., 2011 | 2H | CAAAATTCCGTCTCCATTGT | | ACTTCAACAACAGCGACGAT | | A/C | | Sequencing |  |
| 2_0032 | Druka et al., 2011 | 2H | CAAGTGGAACCCCAGCAA | | CGCCACAGCGTCTGACTC | | A/G | | CAPS |  |
| 2_0039 | Druka et al., 2011 | 2H | CGGAGATGACTCCTCCACA | | CATAAAACGCAAGAGTAAAAA | | T/C | | CAPS |  |
| 2_0390 | Druka et al., 2011 | 2H | AATCTGTTGCTTCCAAGGAG | | AGTGAGGGAATGTAATCGCA | | A/C | | Sequencing |  |
| 2_0417 | Druka et al., 2011 | 2H | AGTATGCTGGTGGATTGCTG | | AACACACGGGCACATAAACC | | T/C | | Sequencing |  |
| 2_0458 | Druka et al., 2011 | 2H | GTCTCCCCATCTTCCTCTGA | | ACGTTATGACAACTGCACCT | | T/C | | CAPS |  |
| 2_0532 | Druka et al., 2011 | 2H | AAGCAGGAGAATTTCCTTCA | | GAGAGGGAAGGGAAGATCAT | | A/C | | CAPS |  |
| 2_0585 | Druka et al., 2011 | 2H | ATATGTAGAGTTGAGCAGCTCTGTCC | | GGACAGTGTAGAAACCTGTAGCTGT | | A/G | | CAPS |  |
| 2_0887 | Druka et al., 2011 | 2H | AAGCGGAACAACACGCAC | | CTGCGACCCCAACGACTC | | A/G | | CAPS |  |
| 2_1286* | Druka et al., 2011 | 2H | GGGGGATTTTATAAGCACAA | | CCTTGGAACCAAAAGTTCAC | | A/G | | Sequencing |  |
| 2_1399 | Druka et al., 2011 | 2H | CGAGGAAGTGAGTTTCTCCT | | GAAATGTTCTTCTCCAAGGA | | T/C | | Sequencing |  |
| 2_0669** | Druka et al., 2011 | 2H | AATCAAGGGAATCAAGATGC | | TTTCTGATCCAAACGGATAA | | - | | - |  |

*Polymorphic markers localized outside the target interval therefore not analyzed in the mapping populations

**Unsuccessful amplification

**Supplemental Table 2.** Genes tested as candidates for *Mat-c* and their respective forward and reverse primers.

| **Gene name** | **Barley MLOC** | **Fragments** | **Forward primer (5’-3’)** | **Reverse primer (5’-3’)** |
| --- | --- | --- | --- | --- |
| *HvWNK1* | HORVU2Hr1G037990 | 1 | GCTTTCCCATTGATCTAATC | AGATTGAGCGTACAAGGATA |
|  |  | 2 | CTTGCTGTCGCTTCTTTCTT | CACACACCAGAATCACAAGA |
|  |  | 3 | CAAATGATGATGGTTCTGTG | ACCAAAGAGTTTAGGCATCA |
|  |  | 4 | TGTCATCTAGTGGTTTGGTT | ATAGCTTAGGCACTTTGCTT |
|  |  |  |  |  |
| *HvELF5* | HORVU2Hr1G056040 | 1 | CTTACGACCAGCCTAAAAGG | AAATTACGGGTGTGTTTCCC |
|  |  | 2 | AAGTAAAGGGTGAGCAACCA | GTGTAGAATCTGCCGAGCTT |
|  |  | 3 | CGTCTGAGCTGCTAAACAAG | CATGTCCACATTCAGTTTGG |
|  |  |  |  |  |
| *HvVIP4* | HORVU2Hr1G063950 | 1 | GCAGAACTCACCTCAGAGAG | GCCTCTGTTTCACTTTCCTC |
|  |  | 2 | AAAACTGGAATTGGTCTTGC | ACTGAATGGATGGAAAAGGA |
|  |  | 3 | TTCTTGCATTTGTTTTGCAT | TTGCTGCATGAAAAATGTTC |
|  |  | 4 | CGTTACAGATGAATCTGGGA | GAGCATATTCAACCACTGGA |
|  |  | 5 | GCATTTTTCAGCGAAGTTTT | TATTCGACTGAGAATGTGCA |
|  |  | 6 | GCTGAAGCACTAGCTGAAAG | GTTACCAGACGATTCTGTGG |
|  |  |  |  |  |
| *HvCEN* | HORVU2Hr1G072750 | 1 | CAATTCCCTACCGCTACATC | TGTACAAGTTCCCCAATCAG |
|  |  | 2 | ATGTGCTCAAATAGTGTGGC | AGCTTGGGTTAGTGGTTACC |
|  |  | 3 | AGCCATCTCGTCTGTACACA | GCAGATGTAGGTTGCACGTA |
|  |  | 4 | CAGCCATACCCTTCTTCTCT | GGAGGAGTCCGTTAAGAGAG |

**Supplemental Table 3.** List of the 129 high-confidence genes located in the 31 Mbp region between the two markers 2_0585 located at bp 494,259,016 on barley chromosome 2H and marker 3_0265 at bp 525,481,435. The *Mat-c* gene has been marked in yellow.

| Gene name | Chromosome | Start | End | Confidence | Annotation |
| --- | --- | --- | --- | --- | --- |
| [HORVU2Hr1G070090.3](https://apex.ipk-gatersleben.de/apex/f?p=284:45:::NO::P45_GENE_NAME:HORVU2Hr1G070090.3) | [chr2H](http://plants.ensembl.org/Hordeum_vulgare/Location/View?r=chr2H:494259016-494295827) | 494259016 | 494295827 | HC_G | 2-oxoglutarate dehydrogenase, E1 component |
| [HORVU2Hr1G070110.8](https://apex.ipk-gatersleben.de/apex/f?p=284:45:::NO::P45_GENE_NAME:HORVU2Hr1G070110.8) | [chr2H](http://plants.ensembl.org/Hordeum_vulgare/Location/View?r=chr2H:494296868-494300587) | 494296868 | 494300587 | HC_G | unknown function |
| [HORVU2Hr1G070140.3](https://apex.ipk-gatersleben.de/apex/f?p=284:45:::NO::P45_GENE_NAME:HORVU2Hr1G070140.3) | [chr2H](http://plants.ensembl.org/Hordeum_vulgare/Location/View?r=chr2H:494444676-494447773) | 494444676 | 494447773 | HC_G | Heavy metal transport/detoxification superfamily protein |
| [HORVU2Hr1G070160.1](https://apex.ipk-gatersleben.de/apex/f?p=284:45:::NO::P45_GENE_NAME:HORVU2Hr1G070160.1) | [chr2H](http://plants.ensembl.org/Hordeum_vulgare/Location/View?r=chr2H:494639515-494641929) | 494639515 | 494641929 | HC_G | Heavy metal transport/detoxification superfamily protein |
| [HORVU2Hr1G070210.1](https://apex.ipk-gatersleben.de/apex/f?p=284:45:::NO::P45_GENE_NAME:HORVU2Hr1G070210.1) | [chr2H](http://plants.ensembl.org/Hordeum_vulgare/Location/View?r=chr2H:495219301-495219549) | 495219301 | 495219549 | HC_G | Single-stranded nucleic acid binding R3H domain protein |
| [HORVU2Hr1G070220.1](https://apex.ipk-gatersleben.de/apex/f?p=284:45:::NO::P45_GENE_NAME:HORVU2Hr1G070220.1) | [chr2H](http://plants.ensembl.org/Hordeum_vulgare/Location/View?r=chr2H:495710328-495711652) | 495710328 | 495711652 | HC_G | ATP synthase subunit a |
| [HORVU2Hr1G070240.1](https://apex.ipk-gatersleben.de/apex/f?p=284:45:::NO::P45_GENE_NAME:HORVU2Hr1G070240.1) | [chr2H](http://plants.ensembl.org/Hordeum_vulgare/Location/View?r=chr2H:495726882-495727141) | 495726882 | 495727141 | HC_G | Cytochrome C assembly protein |
| [HORVU2Hr1G070250.1](https://apex.ipk-gatersleben.de/apex/f?p=284:45:::NO::P45_GENE_NAME:HORVU2Hr1G070250.1) | [chr2H](http://plants.ensembl.org/Hordeum_vulgare/Location/View?r=chr2H:495727677-495727928) | 495727677 | 495727928 | HC_G | Cytochrome c biogenesis Fn |
| [HORVU2Hr1G070280.6](https://apex.ipk-gatersleben.de/apex/f?p=284:45:::NO::P45_GENE_NAME:HORVU2Hr1G070280.6) | [chr2H](http://plants.ensembl.org/Hordeum_vulgare/Location/View?r=chr2H:496098584-496100672) | 496098584 | 496100672 | HC_G | P-loop containing nucleoside triphosphate hydrolases superfamily protein |
| [HORVU2Hr1G070300.1](https://apex.ipk-gatersleben.de/apex/f?p=284:45:::NO::P45_GENE_NAME:HORVU2Hr1G070300.1) | [chr2H](http://plants.ensembl.org/Hordeum_vulgare/Location/View?r=chr2H:496220250-496224886) | 496220250 | 496224886 | HC_G | YELLOW STRIPE like 6 |
| [HORVU2Hr1G070310.2](https://apex.ipk-gatersleben.de/apex/f?p=284:45:::NO::P45_GENE_NAME:HORVU2Hr1G070310.2) | [chr2H](http://plants.ensembl.org/Hordeum_vulgare/Location/View?r=chr2H:496635096-496636060) | 496635096 | 496636060 | HC_G | hydroxysteroid dehydrogenase 1 |
| [HORVU2Hr1G070320.14](https://apex.ipk-gatersleben.de/apex/f?p=284:45:::NO::P45_GENE_NAME:HORVU2Hr1G070320.14) | [chr2H](http://plants.ensembl.org/Hordeum_vulgare/Location/View?r=chr2H:496671166-496675510) | 496671166 | 496675510 | HC_G | YELLOW STRIPE like 6 |
| [HORVU2Hr1G070360.3](https://apex.ipk-gatersleben.de/apex/f?p=284:45:::NO::P45_GENE_NAME:HORVU2Hr1G070360.3) | [chr2H](http://plants.ensembl.org/Hordeum_vulgare/Location/View?r=chr2H:496740343-496742384) | 496740343 | 496742384 | HC_G | hydroxysteroid dehydrogenase 1 |
| [HORVU2Hr1G070400.4](https://apex.ipk-gatersleben.de/apex/f?p=284:45:::NO::P45_GENE_NAME:HORVU2Hr1G070400.4) | [chr2H](http://plants.ensembl.org/Hordeum_vulgare/Location/View?r=chr2H:497137462-497142115) | 497137462 | 497142115 | HC_G | NagB/RpiA/CoA transferase-like superfamily protein |
| [HORVU2Hr1G070410.5](https://apex.ipk-gatersleben.de/apex/f?p=284:45:::NO::P45_GENE_NAME:HORVU2Hr1G070410.5) | [chr2H](http://plants.ensembl.org/Hordeum_vulgare/Location/View?r=chr2H:497144890-497146838) | 497144890 | 497146838 | HC_G | unknown function |
| [HORVU2Hr1G070440.1](https://apex.ipk-gatersleben.de/apex/f?p=284:45:::NO::P45_GENE_NAME:HORVU2Hr1G070440.1) | [chr2H](http://plants.ensembl.org/Hordeum_vulgare/Location/View?r=chr2H:497411992-497413055) | 497411992 | 497413055 | HC_G | ACT domain repeat 4 |
| [HORVU2Hr1G070450.21](https://apex.ipk-gatersleben.de/apex/f?p=284:45:::NO::P45_GENE_NAME:HORVU2Hr1G070450.21) | [chr2H](http://plants.ensembl.org/Hordeum_vulgare/Location/View?r=chr2H:497513037-497517326) | 497513037 | 497517326 | HC_G | Fatty acid oxidation complex subunit alpha |
| [HORVU2Hr1G070480.1](https://apex.ipk-gatersleben.de/apex/f?p=284:45:::NO::P45_GENE_NAME:HORVU2Hr1G070480.1) | [chr2H](http://plants.ensembl.org/Hordeum_vulgare/Location/View?r=chr2H:497624966-497625457) | 497624966 | 497625457 | HC_G | Heat shock 70 kDa protein 16 |
| [HORVU2Hr1G070490.1](https://apex.ipk-gatersleben.de/apex/f?p=284:45:::NO::P45_GENE_NAME:HORVU2Hr1G070490.1) | [chr2H](http://plants.ensembl.org/Hordeum_vulgare/Location/View?r=chr2H:497977646-497978230) | 497977646 | 497978230 | HC_G | Uncultured bacterium extrachromosomal DNA RGI01661 |
| [HORVU2Hr1G070510.1](https://apex.ipk-gatersleben.de/apex/f?p=284:45:::NO::P45_GENE_NAME:HORVU2Hr1G070510.1) | [chr2H](http://plants.ensembl.org/Hordeum_vulgare/Location/View?r=chr2H:497981394-497981546) | 497981394 | 497981546 | HC_G | 30S ribosomal protein S12 |
| [HORVU2Hr1G070520.2](https://apex.ipk-gatersleben.de/apex/f?p=284:45:::NO::P45_GENE_NAME:HORVU2Hr1G070520.2) | [chr2H](http://plants.ensembl.org/Hordeum_vulgare/Location/View?r=chr2H:497983009-497985252) | 497983009 | 497985252 | HC_G | NAD(P)H-quinone oxidoreductase subunit 2 A, chloroplastic |
| [HORVU2Hr1G070530.1](https://apex.ipk-gatersleben.de/apex/f?p=284:45:::NO::P45_GENE_NAME:HORVU2Hr1G070530.1) | [chr2H](http://plants.ensembl.org/Hordeum_vulgare/Location/View?r=chr2H:497989034-497989147) | 497989034 | 497989147 | HC_G | 50S ribosomal protein L2, chloroplastic |
| [HORVU2Hr1G070550.1](https://apex.ipk-gatersleben.de/apex/f?p=284:45:::NO::P45_GENE_NAME:HORVU2Hr1G070550.1) | [chr2H](http://plants.ensembl.org/Hordeum_vulgare/Location/View?r=chr2H:497989820-497990317) | 497989820 | 497990317 | HC_G | 50S ribosomal protein L2, chloroplastic |
| [HORVU2Hr1G070560.1](https://apex.ipk-gatersleben.de/apex/f?p=284:45:::NO::P45_GENE_NAME:HORVU2Hr1G070560.1) | [chr2H](http://plants.ensembl.org/Hordeum_vulgare/Location/View?r=chr2H:497990645-497990860) | 497990645 | 497990860 | HC_G | 30S ribosomal protein S19, chloroplastic |
| [HORVU2Hr1G070570.1](https://apex.ipk-gatersleben.de/apex/f?p=284:45:::NO::P45_GENE_NAME:HORVU2Hr1G070570.1) | [chr2H](http://plants.ensembl.org/Hordeum_vulgare/Location/View?r=chr2H:497990945-497992121) | 497990945 | 497992121 | HC_G | Photosystem II protein D1 |
| [HORVU2Hr1G070580.1](https://apex.ipk-gatersleben.de/apex/f?p=284:45:::NO::P45_GENE_NAME:HORVU2Hr1G070580.1) | [chr2H](http://plants.ensembl.org/Hordeum_vulgare/Location/View?r=chr2H:497992576-497994200) | 497992576 | 497994200 | HC_G | Maturase K |
| [HORVU2Hr1G070600.1](https://apex.ipk-gatersleben.de/apex/f?p=284:45:::NO::P45_GENE_NAME:HORVU2Hr1G070600.1) | [chr2H](http://plants.ensembl.org/Hordeum_vulgare/Location/View?r=chr2H:497997621-497997806) | 497997621 | 497997806 | HC_G | Photosystem II reaction center protein K |
| [HORVU2Hr1G070670.2](https://apex.ipk-gatersleben.de/apex/f?p=284:45:::NO::P45_GENE_NAME:HORVU2Hr1G070670.2) | [chr2H](http://plants.ensembl.org/Hordeum_vulgare/Location/View?r=chr2H:498695027-498697409) | 498695027 | 498697409 | HC_G | membrane-anchored ubiquitin-fold protein 2 |
| [HORVU2Hr1G070680.1](https://apex.ipk-gatersleben.de/apex/f?p=284:45:::NO::P45_GENE_NAME:HORVU2Hr1G070680.1) | [chr2H](http://plants.ensembl.org/Hordeum_vulgare/Location/View?r=chr2H:499083861-499086294) | 499083861 | 499086294 | HC_G | Betaine aldehyde dehydrogenase 2 |
| [HORVU2Hr1G070690.2](https://apex.ipk-gatersleben.de/apex/f?p=284:45:::NO::P45_GENE_NAME:HORVU2Hr1G070690.2) | [chr2H](http://plants.ensembl.org/Hordeum_vulgare/Location/View?r=chr2H:499985960-499990833) | 499985960 | 499990833 | HC_G | Protein kinase superfamily protein |
| [HORVU2Hr1G070700.1](https://apex.ipk-gatersleben.de/apex/f?p=284:45:::NO::P45_GENE_NAME:HORVU2Hr1G070700.1) | [chr2H](http://plants.ensembl.org/Hordeum_vulgare/Location/View?r=chr2H:500143835-500146439) | 500143835 | 500146439 | HC_G | Glycerophosphodiester phosphodiesterase GDE1 |
| [HORVU2Hr1G070720.25](https://apex.ipk-gatersleben.de/apex/f?p=284:45:::NO::P45_GENE_NAME:HORVU2Hr1G070720.25) | [chr2H](http://plants.ensembl.org/Hordeum_vulgare/Location/View?r=chr2H:500425196-500433096) | 500425196 | 500433096 | HC_G | hydroxyproline-rich glycoprotein family protein |
| [HORVU2Hr1G070800.3](https://apex.ipk-gatersleben.de/apex/f?p=284:45:::NO::P45_GENE_NAME:HORVU2Hr1G070800.3) | [chr2H](http://plants.ensembl.org/Hordeum_vulgare/Location/View?r=chr2H:500802852-500808384) | 500802852 | 500808384 | HC_G | Transport inhibitor response 1-like protein |
| [HORVU2Hr1G070850.2](https://apex.ipk-gatersleben.de/apex/f?p=284:45:::NO::P45_GENE_NAME:HORVU2Hr1G070850.2) | [chr2H](http://plants.ensembl.org/Hordeum_vulgare/Location/View?r=chr2H:501256796-501270695) | 501256796 | 501270695 | HC_G | Phosphoribosylformylglycinamidine synthase |
| [HORVU2Hr1G070890.1](https://apex.ipk-gatersleben.de/apex/f?p=284:45:::NO::P45_GENE_NAME:HORVU2Hr1G070890.1) | [chr2H](http://plants.ensembl.org/Hordeum_vulgare/Location/View?r=chr2H:501486543-501502072) | 501486543 | 501502072 | HC_G | CCA-adding enzyme |
| [HORVU2Hr1G070880.11](https://apex.ipk-gatersleben.de/apex/f?p=284:45:::NO::P45_GENE_NAME:HORVU2Hr1G070880.11) | [chr2H](http://plants.ensembl.org/Hordeum_vulgare/Location/View?r=chr2H:501487374-501489419) | 501487374 | 501489419 | HC_G | Protein TIFY 9 |
| [HORVU2Hr1G070910.1](https://apex.ipk-gatersleben.de/apex/f?p=284:45:::NO::P45_GENE_NAME:HORVU2Hr1G070910.1) | [chr2H](http://plants.ensembl.org/Hordeum_vulgare/Location/View?r=chr2H:501508398-501509024) | 501508398 | 501509024 | HC_G | Serine/threonine-protein phosphatase 7 long form-like protein |
| [HORVU2Hr1G070920.1](https://apex.ipk-gatersleben.de/apex/f?p=284:45:::NO::P45_GENE_NAME:HORVU2Hr1G070920.1) | [chr2H](http://plants.ensembl.org/Hordeum_vulgare/Location/View?r=chr2H:501509207-501509347) | 501509207 | 501509347 | HC_G | Serine/threonine-protein phosphatase 7 long form-like protein |
| [HORVU2Hr1G070940.10](https://apex.ipk-gatersleben.de/apex/f?p=284:45:::NO::P45_GENE_NAME:HORVU2Hr1G070940.10) | [chr2H](http://plants.ensembl.org/Hordeum_vulgare/Location/View?r=chr2H:502010441-502020744) | 502010441 | 502020744 | HC_G | RING finger protein |
| [HORVU2Hr1G070980.1](https://apex.ipk-gatersleben.de/apex/f?p=284:45:::NO::P45_GENE_NAME:HORVU2Hr1G070980.1) | [chr2H](http://plants.ensembl.org/Hordeum_vulgare/Location/View?r=chr2H:502687724-502688440) | 502687724 | 502688440 | HC_G | S-adenosyl-L-methionine-dependent methyltransferases superfamily protein |
| [HORVU2Hr1G070990.1](https://apex.ipk-gatersleben.de/apex/f?p=284:45:::NO::P45_GENE_NAME:HORVU2Hr1G070990.1) | [chr2H](http://plants.ensembl.org/Hordeum_vulgare/Location/View?r=chr2H:502852036-502853946) | 502852036 | 502853946 | HC_G | LRR receptor-like serine/threonine-protein kinase EFR |
| [HORVU2Hr1G071040.1](https://apex.ipk-gatersleben.de/apex/f?p=284:45:::NO::P45_GENE_NAME:HORVU2Hr1G071040.1) | [chr2H](http://plants.ensembl.org/Hordeum_vulgare/Location/View?r=chr2H:503508616-503508855) | 503508616 | 503508855 | HC_G | Flavin-containing monooxygenase |
| [HORVU2Hr1G071070.4](https://apex.ipk-gatersleben.de/apex/f?p=284:45:::NO::P45_GENE_NAME:HORVU2Hr1G071070.4) | [chr2H](http://plants.ensembl.org/Hordeum_vulgare/Location/View?r=chr2H:503661101-503664140) | 503661101 | 503664140 | HC_G | Carboxypeptidase Y homolog A |
| [HORVU2Hr1G071080.1](https://apex.ipk-gatersleben.de/apex/f?p=284:45:::NO::P45_GENE_NAME:HORVU2Hr1G071080.1) | [chr2H](http://plants.ensembl.org/Hordeum_vulgare/Location/View?r=chr2H:503879648-503887381) | 503879648 | 503887381 | HC_G | Chaperone protein ClpB |
| [HORVU2Hr1G071100.8](https://apex.ipk-gatersleben.de/apex/f?p=284:45:::NO::P45_GENE_NAME:HORVU2Hr1G071100.8) | [chr2H](http://plants.ensembl.org/Hordeum_vulgare/Location/View?r=chr2H:504798542-504801556) | 504798542 | 504801556 | HC_G | Myb/SANT-like DNA-binding domain protein |
| [HORVU2Hr1G071120.1](https://apex.ipk-gatersleben.de/apex/f?p=284:45:::NO::P45_GENE_NAME:HORVU2Hr1G071120.1) | [chr2H](http://plants.ensembl.org/Hordeum_vulgare/Location/View?r=chr2H:505114609-505115478) | 505114609 | 505115478 | HC_G | Pectin acetylesterase 10 |
| [HORVU2Hr1G071130.1](https://apex.ipk-gatersleben.de/apex/f?p=284:45:::NO::P45_GENE_NAME:HORVU2Hr1G071130.1) | [chr2H](http://plants.ensembl.org/Hordeum_vulgare/Location/View?r=chr2H:505135060-505135577) | 505135060 | 505135577 | HC_G | Esterase/lipase/thioesterase family protein |
| [HORVU2Hr1G071160.1](https://apex.ipk-gatersleben.de/apex/f?p=284:45:::NO::P45_GENE_NAME:HORVU2Hr1G071160.1) | [chr2H](http://plants.ensembl.org/Hordeum_vulgare/Location/View?r=chr2H:505549572-505549717) | 505549572 | 505549717 | HC_G | Succinyl-CoA ligase [ADP-forming] subunit alpha-1, mitochondrial |
| [HORVU2Hr1G071170.4](https://apex.ipk-gatersleben.de/apex/f?p=284:45:::NO::P45_GENE_NAME:HORVU2Hr1G071170.4) | [chr2H](http://plants.ensembl.org/Hordeum_vulgare/Location/View?r=chr2H:505635954-505638188) | 505635954 | 505638188 | HC_G | Centromere protein S |
| [HORVU2Hr1G071180.1](https://apex.ipk-gatersleben.de/apex/f?p=284:45:::NO::P45_GENE_NAME:HORVU2Hr1G071180.1) | [chr2H](http://plants.ensembl.org/Hordeum_vulgare/Location/View?r=chr2H:505643000-505644750) | 505643000 | 505644750 | HC_G | Beta-fructofuranosidase, insoluble isoenzyme 4 |
| [HORVU2Hr1G071210.1](https://apex.ipk-gatersleben.de/apex/f?p=284:45:::NO::P45_GENE_NAME:HORVU2Hr1G071210.1) | [chr2H](http://plants.ensembl.org/Hordeum_vulgare/Location/View?r=chr2H:505995413-505995953) | 505995413 | 505995953 | HC_G | Cytochrome c oxidase biogenesis protein Cmc1-like |
| [HORVU2Hr1G071220.1](https://apex.ipk-gatersleben.de/apex/f?p=284:45:::NO::P45_GENE_NAME:HORVU2Hr1G071220.1) | [chr2H](http://plants.ensembl.org/Hordeum_vulgare/Location/View?r=chr2H:506016030-506019312) | 506016030 | 506019312 | HC_G | transferases, transferring glycosyl groups |
| [HORVU2Hr1G071240.12](https://apex.ipk-gatersleben.de/apex/f?p=284:45:::NO::P45_GENE_NAME:HORVU2Hr1G071240.12) | [chr2H](http://plants.ensembl.org/Hordeum_vulgare/Location/View?r=chr2H:507109397-507115872) | 507109397 | 507115872 | HC_G | MEI2-like protein 1 |
| [HORVU2Hr1G071250.1](https://apex.ipk-gatersleben.de/apex/f?p=284:45:::NO::P45_GENE_NAME:HORVU2Hr1G071250.1) | [chr2H](http://plants.ensembl.org/Hordeum_vulgare/Location/View?r=chr2H:507161548-507162944) | 507161548 | 507162944 | HC_G | RING/U-box superfamily protein |
| [HORVU2Hr1G071260.1](https://apex.ipk-gatersleben.de/apex/f?p=284:45:::NO::P45_GENE_NAME:HORVU2Hr1G071260.1) | [chr2H](http://plants.ensembl.org/Hordeum_vulgare/Location/View?r=chr2H:507163779-507165180) | 507163779 | 507165180 | HC_G | 1,4-dihydroxy-2-naphthoate phytyltransferase |
| [HORVU2Hr1G071270.4](https://apex.ipk-gatersleben.de/apex/f?p=284:45:::NO::P45_GENE_NAME:HORVU2Hr1G071270.4) | [chr2H](http://plants.ensembl.org/Hordeum_vulgare/Location/View?r=chr2H:507785971-507787466) | 507785971 | 507787466 | HC_G | Ethylene-responsive transcription factor 1 |
| [HORVU2Hr1G071310.1](https://apex.ipk-gatersleben.de/apex/f?p=284:45:::NO::P45_GENE_NAME:HORVU2Hr1G071310.1) | [chr2H](http://plants.ensembl.org/Hordeum_vulgare/Location/View?r=chr2H:507896155-507898070) | 507896155 | 507898070 | HC_G | Apocytochrome f |
| [HORVU2Hr1G071330.6](https://apex.ipk-gatersleben.de/apex/f?p=284:45:::NO::P45_GENE_NAME:HORVU2Hr1G071330.6) | [chr2H](http://plants.ensembl.org/Hordeum_vulgare/Location/View?r=chr2H:508786030-508794435) | 508786030 | 508794435 | HC_G | Glycine--tRNA ligase |
| [HORVU2Hr1G071350.1](https://apex.ipk-gatersleben.de/apex/f?p=284:45:::NO::P45_GENE_NAME:HORVU2Hr1G071350.1) | [chr2H](http://plants.ensembl.org/Hordeum_vulgare/Location/View?r=chr2H:509053603-509056014) | 509053603 | 509056014 | HC_G | Cytochrome b-c1 complex subunit Rieske, mitochondrial |
| [HORVU2Hr1G071380.1](https://apex.ipk-gatersleben.de/apex/f?p=284:45:::NO::P45_GENE_NAME:HORVU2Hr1G071380.1) | [chr2H](http://plants.ensembl.org/Hordeum_vulgare/Location/View?r=chr2H:509207349-509210834) | 509207349 | 509210834 | HC_G | Exostosin family protein |
| [HORVU2Hr1G071410.1](https://apex.ipk-gatersleben.de/apex/f?p=284:45:::NO::P45_GENE_NAME:HORVU2Hr1G071410.1) | [chr2H](http://plants.ensembl.org/Hordeum_vulgare/Location/View?r=chr2H:509477352-509478839) | 509477352 | 509478839 | HC_G | Thioredoxin-like protein 4B |
| [HORVU2Hr1G071420.1](https://apex.ipk-gatersleben.de/apex/f?p=284:45:::NO::P45_GENE_NAME:HORVU2Hr1G071420.1) | [chr2H](http://plants.ensembl.org/Hordeum_vulgare/Location/View?r=chr2H:509762112-509762628) | 509762112 | 509762628 | HC_G | Pumilio domain-containing protein |
| [HORVU2Hr1G071430.1](https://apex.ipk-gatersleben.de/apex/f?p=284:45:::NO::P45_GENE_NAME:HORVU2Hr1G071430.1) | [chr2H](http://plants.ensembl.org/Hordeum_vulgare/Location/View?r=chr2H:509763228-509763419) | 509763228 | 509763419 | HC_G | ATP binding microtubule motor family protein |
| [HORVU2Hr1G071440.2](https://apex.ipk-gatersleben.de/apex/f?p=284:45:::NO::P45_GENE_NAME:HORVU2Hr1G071440.2) | [chr2H](http://plants.ensembl.org/Hordeum_vulgare/Location/View?r=chr2H:509767245-509767689) | 509767245 | 509767689 | HC_G | unknown function |
| [HORVU2Hr1G071450.2](https://apex.ipk-gatersleben.de/apex/f?p=284:45:::NO::P45_GENE_NAME:HORVU2Hr1G071450.2) | [chr2H](http://plants.ensembl.org/Hordeum_vulgare/Location/View?r=chr2H:509780488-509782854) | 509780488 | 509782854 | HC_G | nudix hydrolase homolog 13 |
| [HORVU2Hr1G071470.1](https://apex.ipk-gatersleben.de/apex/f?p=284:45:::NO::P45_GENE_NAME:HORVU2Hr1G071470.1) | [chr2H](http://plants.ensembl.org/Hordeum_vulgare/Location/View?r=chr2H:510090640-510091987) | 510090640 | 510091987 | HC_G | Ethylene-responsive transcription factor 8 |
| [HORVU2Hr1G071530.2](https://apex.ipk-gatersleben.de/apex/f?p=284:45:::NO::P45_GENE_NAME:HORVU2Hr1G071530.2) | [chr2H](http://plants.ensembl.org/Hordeum_vulgare/Location/View?r=chr2H:510201858-510203426) | 510201858 | 510203426 | HC_G | Pentatricopeptide repeat-containing protein |
| [HORVU2Hr1G071540.16](https://apex.ipk-gatersleben.de/apex/f?p=284:45:::NO::P45_GENE_NAME:HORVU2Hr1G071540.16) | [chr2H](http://plants.ensembl.org/Hordeum_vulgare/Location/View?r=chr2H:510205399-510223432) | 510205399 | 510223432 | HC_G | 5'-AMP-activated protein kinase subunit gamma-1 |
| [HORVU2Hr1G071570.17](https://apex.ipk-gatersleben.de/apex/f?p=284:45:::NO::P45_GENE_NAME:HORVU2Hr1G071570.17) | [chr2H](http://plants.ensembl.org/Hordeum_vulgare/Location/View?r=chr2H:510426276-510431354) | 510426276 | 510431354 | HC_G | Potassium transporter family protein |
| [HORVU2Hr1G071630.24](https://apex.ipk-gatersleben.de/apex/f?p=284:45:::NO::P45_GENE_NAME:HORVU2Hr1G071630.24) | [chr2H](http://plants.ensembl.org/Hordeum_vulgare/Location/View?r=chr2H:511370830-511373867) | 511370830 | 511373867 | HC_G | Potassium transporter family protein |
| [HORVU2Hr1G071670.10](https://apex.ipk-gatersleben.de/apex/f?p=284:45:::NO::P45_GENE_NAME:HORVU2Hr1G071670.10) | [chr2H](http://plants.ensembl.org/Hordeum_vulgare/Location/View?r=chr2H:511432484-511435975) | 511432484 | 511435975 | HC_G | DNA cross-link repair 1A protein |
| [HORVU2Hr1G071710.5](https://apex.ipk-gatersleben.de/apex/f?p=284:45:::NO::P45_GENE_NAME:HORVU2Hr1G071710.5) | [chr2H](http://plants.ensembl.org/Hordeum_vulgare/Location/View?r=chr2H:511663421-511673912) | 511663421 | 511673912 | HC_G | tyrosyl-DNA phosphodiesterase-related |
| [HORVU2Hr1G071780.1](https://apex.ipk-gatersleben.de/apex/f?p=284:45:::NO::P45_GENE_NAME:HORVU2Hr1G071780.1) | [chr2H](http://plants.ensembl.org/Hordeum_vulgare/Location/View?r=chr2H:512543636-512545785) | 512543636 | 512545785 | HC_G | SBP (S-ribonuclease binding protein) family protein |
| [HORVU2Hr1G071830.18](https://apex.ipk-gatersleben.de/apex/f?p=284:45:::NO::P45_GENE_NAME:HORVU2Hr1G071830.18) | [chr2H](http://plants.ensembl.org/Hordeum_vulgare/Location/View?r=chr2H:513179516-513200801) | 513179516 | 513200801 | HC_G | Cysteine proteinases superfamily protein |
| [HORVU2Hr1G071850.6](https://apex.ipk-gatersleben.de/apex/f?p=284:45:::NO::P45_GENE_NAME:HORVU2Hr1G071850.6) | [chr2H](http://plants.ensembl.org/Hordeum_vulgare/Location/View?r=chr2H:513414227-513418176) | 513414227 | 513418176 | HC_G | Protein phosphatase 2C family protein |
| [HORVU2Hr1G071860.2](https://apex.ipk-gatersleben.de/apex/f?p=284:45:::NO::P45_GENE_NAME:HORVU2Hr1G071860.2) | [chr2H](http://plants.ensembl.org/Hordeum_vulgare/Location/View?r=chr2H:513418749-513425246) | 513418749 | 513425246 | HC_G | NAD(P)-binding Rossmann-fold superfamily protein |
| [HORVU2Hr1G071880.2](https://apex.ipk-gatersleben.de/apex/f?p=284:45:::NO::P45_GENE_NAME:HORVU2Hr1G071880.2) | [chr2H](http://plants.ensembl.org/Hordeum_vulgare/Location/View?r=chr2H:513917440-513919304) | 513917440 | 513919304 | HC_G | OJ000315_02.4 protein |
| [HORVU2Hr1G071890.1](https://apex.ipk-gatersleben.de/apex/f?p=284:45:::NO::P45_GENE_NAME:HORVU2Hr1G071890.1) | [chr2H](http://plants.ensembl.org/Hordeum_vulgare/Location/View?r=chr2H:514559374-514560871) | 514559374 | 514560871 | HC_G | Desiccation-related protein PCC13-62 |
| [HORVU2Hr1G071930.3](https://apex.ipk-gatersleben.de/apex/f?p=284:45:::NO::P45_GENE_NAME:HORVU2Hr1G071930.3) | [chr2H](http://plants.ensembl.org/Hordeum_vulgare/Location/View?r=chr2H:515060435-515061557) | 515060435 | 515061557 | HC_G | heme oxygenase 4 |
| [HORVU2Hr1G071940.1](https://apex.ipk-gatersleben.de/apex/f?p=284:45:::NO::P45_GENE_NAME:HORVU2Hr1G071940.1) | [chr2H](http://plants.ensembl.org/Hordeum_vulgare/Location/View?r=chr2H:515219576-515219951) | 515219576 | 515219951 | HC_G | Photosystem II stability/assembly factor HCF136 |
| [HORVU2Hr1G071960.2](https://apex.ipk-gatersleben.de/apex/f?p=284:45:::NO::P45_GENE_NAME:HORVU2Hr1G071960.2) | [chr2H](http://plants.ensembl.org/Hordeum_vulgare/Location/View?r=chr2H:515420936-515427029) | 515420936 | 515427029 | HC_G | Acetyl-coenzyme A synthetase |
| [HORVU2Hr1G071980.2](https://apex.ipk-gatersleben.de/apex/f?p=284:45:::NO::P45_GENE_NAME:HORVU2Hr1G071980.2) | [chr2H](http://plants.ensembl.org/Hordeum_vulgare/Location/View?r=chr2H:515568411-515580494) | 515568411 | 515580494 | HC_G | Heparan-alpha-glucosaminide N-acetyltransferase |
| [HORVU2Hr1G072010.1](https://apex.ipk-gatersleben.de/apex/f?p=284:45:::NO::P45_GENE_NAME:HORVU2Hr1G072010.1) | [chr2H](http://plants.ensembl.org/Hordeum_vulgare/Location/View?r=chr2H:515704629-515708198) | 515704629 | 515708198 | HC_G | Mitochondrial import inner membrane translocase subunit Tim17/Tim22/Tim23 family protein |
| [HORVU2Hr1G072020.8](https://apex.ipk-gatersleben.de/apex/f?p=284:45:::NO::P45_GENE_NAME:HORVU2Hr1G072020.8) | [chr2H](http://plants.ensembl.org/Hordeum_vulgare/Location/View?r=chr2H:515709643-515715973) | 515709643 | 515715973 | HC_G | Chaperone protein ClpB |
| [HORVU2Hr1G072040.3](https://apex.ipk-gatersleben.de/apex/f?p=284:45:::NO::P45_GENE_NAME:HORVU2Hr1G072040.3) | [chr2H](http://plants.ensembl.org/Hordeum_vulgare/Location/View?r=chr2H:515784467-515785278) | 515784467 | 515785278 | HC_G | Short-chain dehydrogenase reductase 2a |
| [HORVU2Hr1G072050.1](https://apex.ipk-gatersleben.de/apex/f?p=284:45:::NO::P45_GENE_NAME:HORVU2Hr1G072050.1) | [chr2H](http://plants.ensembl.org/Hordeum_vulgare/Location/View?r=chr2H:516117796-516118002) | 516117796 | 516118002 | HC_G | DNA repair protein rhp54 |
| [HORVU2Hr1G072100.1](https://apex.ipk-gatersleben.de/apex/f?p=284:45:::NO::P45_GENE_NAME:HORVU2Hr1G072100.1) | [chr2H](http://plants.ensembl.org/Hordeum_vulgare/Location/View?r=chr2H:516257605-516266365) | 516257605 | 516266365 | HC_G | UDP-Glycosyltransferase superfamily protein |
| [HORVU2Hr1G072100.1](https://apex.ipk-gatersleben.de/apex/f?p=284:45:::NO::P45_GENE_NAME:HORVU2Hr1G072100.1) | [chr2H](http://plants.ensembl.org/Hordeum_vulgare/Location/View?r=chr2H:516257605-516266365) | 516257605 | 516266365 | HC_G | UDP-Glycosyltransferase superfamily protein |
| [HORVU2Hr1G072110.1](https://apex.ipk-gatersleben.de/apex/f?p=284:45:::NO::P45_GENE_NAME:HORVU2Hr1G072110.1) | [chr2H](http://plants.ensembl.org/Hordeum_vulgare/Location/View?r=chr2H:516269822-516270314) | 516269822 | 516270314 | HC_G | Intracellular protease, PfpI family |
| [HORVU2Hr1G072140.5](https://apex.ipk-gatersleben.de/apex/f?p=284:45:::NO::P45_GENE_NAME:HORVU2Hr1G072140.5) | [chr2H](http://plants.ensembl.org/Hordeum_vulgare/Location/View?r=chr2H:516578497-516583796) | 516578497 | 516583796 | HC_G | Uridylate kinase |
| [HORVU2Hr1G072180.1](https://apex.ipk-gatersleben.de/apex/f?p=284:45:::NO::P45_GENE_NAME:HORVU2Hr1G072180.1) | [chr2H](http://plants.ensembl.org/Hordeum_vulgare/Location/View?r=chr2H:516922164-516923941) | 516922164 | 516923941 | HC_G | prephenate dehydratase 1 |
| [HORVU2Hr1G072210.3](https://apex.ipk-gatersleben.de/apex/f?p=284:45:::NO::P45_GENE_NAME:HORVU2Hr1G072210.3) | [chr2H](http://plants.ensembl.org/Hordeum_vulgare/Location/View?r=chr2H:517227991-517229981) | 517227991 | 517229981 | HC_G | Arogenate dehydratase/prephenate dehydratase 6, chloroplastic |
| [HORVU2Hr1G072220.7](https://apex.ipk-gatersleben.de/apex/f?p=284:45:::NO::P45_GENE_NAME:HORVU2Hr1G072220.7) | [chr2H](http://plants.ensembl.org/Hordeum_vulgare/Location/View?r=chr2H:517597906-517601149) | 517597906 | 517601149 | HC_G | histidinol dehydrogenase |
| [HORVU2Hr1G072230.2](https://apex.ipk-gatersleben.de/apex/f?p=284:45:::NO::P45_GENE_NAME:HORVU2Hr1G072230.2) | [chr2H](http://plants.ensembl.org/Hordeum_vulgare/Location/View?r=chr2H:517909440-517911157) | 517909440 | 517911157 | HC_G | Auxin response factor 13 |
| [HORVU2Hr1G072290.2](https://apex.ipk-gatersleben.de/apex/f?p=284:45:::NO::P45_GENE_NAME:HORVU2Hr1G072290.2) | [chr2H](http://plants.ensembl.org/Hordeum_vulgare/Location/View?r=chr2H:518266983-518269023) | 518266983 | 518269023 | HC_G | Histone-lysine N-methyltransferase |
| [HORVU2Hr1G072400.1](https://apex.ipk-gatersleben.de/apex/f?p=284:45:::NO::P45_GENE_NAME:HORVU2Hr1G072400.1) | [chr2H](http://plants.ensembl.org/Hordeum_vulgare/Location/View?r=chr2H:519108149-519110415) | 519108149 | 519110415 | HC_G | Cytochrome P450 superfamily protein |
| [HORVU2Hr1G072410.1](https://apex.ipk-gatersleben.de/apex/f?p=284:45:::NO::P45_GENE_NAME:HORVU2Hr1G072410.1) | [chr2H](http://plants.ensembl.org/Hordeum_vulgare/Location/View?r=chr2H:519493424-519494835) | 519493424 | 519494835 | HC_G | unknown function |
| [HORVU2Hr1G072420.11](https://apex.ipk-gatersleben.de/apex/f?p=284:45:::NO::P45_GENE_NAME:HORVU2Hr1G072420.11) | [chr2H](http://plants.ensembl.org/Hordeum_vulgare/Location/View?r=chr2H:519528221-519529717) | 519528221 | 519529717 | HC_G | Family of unknown function (DUF662) |
| [HORVU2Hr1G072450.1](https://apex.ipk-gatersleben.de/apex/f?p=284:45:::NO::P45_GENE_NAME:HORVU2Hr1G072450.1) | [chr2H](http://plants.ensembl.org/Hordeum_vulgare/Location/View?r=chr2H:519644151-519644538) | 519644151 | 519644538 | HC_G | cysteine synthase D2 |
| [HORVU2Hr1G072470.2](https://apex.ipk-gatersleben.de/apex/f?p=284:45:::NO::P45_GENE_NAME:HORVU2Hr1G072470.2) | [chr2H](http://plants.ensembl.org/Hordeum_vulgare/Location/View?r=chr2H:520085856-520092112) | 520085856 | 520092112 | HC_G | RNA polymerase II C-terminal domain phosphatase-like 4 |
| [HORVU2Hr1G072490.2](https://apex.ipk-gatersleben.de/apex/f?p=284:45:::NO::P45_GENE_NAME:HORVU2Hr1G072490.2) | [chr2H](http://plants.ensembl.org/Hordeum_vulgare/Location/View?r=chr2H:520168486-520169282) | 520168486 | 520169282 | HC_G | Starch branching enzyme 2 |
| [HORVU2Hr1G072500.34](https://apex.ipk-gatersleben.de/apex/f?p=284:45:::NO::P45_GENE_NAME:HORVU2Hr1G072500.34) | [chr2H](http://plants.ensembl.org/Hordeum_vulgare/Location/View?r=chr2H:520252636-520265966) | 520252636 | 520265966 | HC_G | 1,4-alpha-glucan branching enzyme GlgB |
| [HORVU2Hr1G072510.1](https://apex.ipk-gatersleben.de/apex/f?p=284:45:::NO::P45_GENE_NAME:HORVU2Hr1G072510.1) | [chr2H](http://plants.ensembl.org/Hordeum_vulgare/Location/View?r=chr2H:520255665-520255964) | 520255665 | 520255964 | HC_G | Myosin-J heavy chain |
| [HORVU2Hr1G072530.1](https://apex.ipk-gatersleben.de/apex/f?p=284:45:::NO::P45_GENE_NAME:HORVU2Hr1G072530.1) | [chr2H](http://plants.ensembl.org/Hordeum_vulgare/Location/View?r=chr2H:520437130-520445164) | 520437130 | 520445164 | HC_G | Calcineurin-like metallo-phosphoesterase superfamily protein |
| [HORVU2Hr1G072570.5](https://apex.ipk-gatersleben.de/apex/f?p=284:45:::NO::P45_GENE_NAME:HORVU2Hr1G072570.5) | [chr2H](http://plants.ensembl.org/Hordeum_vulgare/Location/View?r=chr2H:520630762-520633269) | 520630762 | 520633269 | HC_G | histone deacetylase 9 |
| [HORVU2Hr1G072590.1](https://apex.ipk-gatersleben.de/apex/f?p=284:45:::NO::P45_GENE_NAME:HORVU2Hr1G072590.1) | [chr2H](http://plants.ensembl.org/Hordeum_vulgare/Location/View?r=chr2H:520771797-520779116) | 520771797 | 520779116 | HC_G | alkaline/neutral invertase |
| [HORVU2Hr1G072580.3](https://apex.ipk-gatersleben.de/apex/f?p=284:45:::NO::P45_GENE_NAME:HORVU2Hr1G072580.3) | [chr2H](http://plants.ensembl.org/Hordeum_vulgare/Location/View?r=chr2H:520771905-520775538) | 520771905 | 520775538 | HC_G | Protein kinase superfamily protein |
| [HORVU2Hr1G072600.11](https://apex.ipk-gatersleben.de/apex/f?p=284:45:::NO::P45_GENE_NAME:HORVU2Hr1G072600.11) | [chr2H](http://plants.ensembl.org/Hordeum_vulgare/Location/View?r=chr2H:520779631-520781887) | 520779631 | 520781887 | HC_G | histone deacetylase 9 |
| [HORVU2Hr1G072620.1](https://apex.ipk-gatersleben.de/apex/f?p=284:45:::NO::P45_GENE_NAME:HORVU2Hr1G072620.1) | [chr2H](http://plants.ensembl.org/Hordeum_vulgare/Location/View?r=chr2H:521075842-521076254) | 521075842 | 521076254 | HC_G | Nucleotide-diphospho-sugar transferase domain protein |
| [HORVU2Hr1G072630.1](https://apex.ipk-gatersleben.de/apex/f?p=284:45:::NO::P45_GENE_NAME:HORVU2Hr1G072630.1) | [chr2H](http://plants.ensembl.org/Hordeum_vulgare/Location/View?r=chr2H:521178028-521179283) | 521178028 | 521179283 | HC_G | Major facilitator superfamily protein |
| [HORVU2Hr1G072650.1](https://apex.ipk-gatersleben.de/apex/f?p=284:45:::NO::P45_GENE_NAME:HORVU2Hr1G072650.1) | [chr2H](http://plants.ensembl.org/Hordeum_vulgare/Location/View?r=chr2H:521292906-521295057) | 521292906 | 521295057 | HC_G | ATP synthase D chain, mitochondrial |
| [HORVU2Hr1G072660.2](https://apex.ipk-gatersleben.de/apex/f?p=284:45:::NO::P45_GENE_NAME:HORVU2Hr1G072660.2) | [chr2H](http://plants.ensembl.org/Hordeum_vulgare/Location/View?r=chr2H:521460787-521462956) | 521460787 | 521462956 | HC_G | ATP synthase D chain, mitochondrial |
| [HORVU2Hr1G072670.2](https://apex.ipk-gatersleben.de/apex/f?p=284:45:::NO::P45_GENE_NAME:HORVU2Hr1G072670.2) | [chr2H](http://plants.ensembl.org/Hordeum_vulgare/Location/View?r=chr2H:521539172-521540671) | 521539172 | 521540671 | HC_G | Cytochrome P450 superfamily protein |
| [HORVU2Hr1G072680.1](https://apex.ipk-gatersleben.de/apex/f?p=284:45:::NO::P45_GENE_NAME:HORVU2Hr1G072680.1) | [chr2H](http://plants.ensembl.org/Hordeum_vulgare/Location/View?r=chr2H:521647166-521647680) | 521647166 | 521647680 | HC_G | RuBisCO large subunit-binding protein subunit beta, chloroplastic |
| [HORVU2Hr1G072690.2](https://apex.ipk-gatersleben.de/apex/f?p=284:45:::NO::P45_GENE_NAME:HORVU2Hr1G072690.2) | [chr2H](http://plants.ensembl.org/Hordeum_vulgare/Location/View?r=chr2H:521773382-521774984) | 521773382 | 521774984 | HC_G | purple acid phosphatase 22 |
| [HORVU2Hr1G072700.1](https://apex.ipk-gatersleben.de/apex/f?p=284:45:::NO::P45_GENE_NAME:HORVU2Hr1G072700.1) | [chr2H](http://plants.ensembl.org/Hordeum_vulgare/Location/View?r=chr2H:522005122-522008100) | 522005122 | 522008100 | HC_G | purple acid phosphatase 22 |
| [HORVU2Hr1G072730.1](https://apex.ipk-gatersleben.de/apex/f?p=284:45:::NO::P45_GENE_NAME:HORVU2Hr1G072730.1) | [chr2H](http://plants.ensembl.org/Hordeum_vulgare/Location/View?r=chr2H:523338401-523339409) | 523338401 | 523339409 | HC_G | HVA22-like protein G |
| [HORVU2Hr1G072740.1](https://apex.ipk-gatersleben.de/apex/f?p=284:45:::NO::P45_GENE_NAME:HORVU2Hr1G072740.1) | [chr2H](http://plants.ensembl.org/Hordeum_vulgare/Location/View?r=chr2H:523358478-523359486) | 523358478 | 523359486 | HC_G | HVA22-like protein G |
| [HORVU2Hr1G072750.4](https://apex.ipk-gatersleben.de/apex/f?p=284:45:::NO::P45_GENE_NAME:HORVU2Hr1G072750.4) | [chr2H](http://plants.ensembl.org/Hordeum_vulgare/Location/View?r=chr2H:523377523-523379139) | 523377523 | 523379139 | HC_G | Protein TERMINAL FLOWER 1 |
| [HORVU2Hr1G072830.1](https://apex.ipk-gatersleben.de/apex/f?p=284:45:::NO::P45_GENE_NAME:HORVU2Hr1G072830.1) | [chr2H](http://plants.ensembl.org/Hordeum_vulgare/Location/View?r=chr2H:524441151-524447848) | 524441151 | 524447848 | HC_G | alpha/beta-Hydrolases superfamily protein |
| [HORVU2Hr1G072850.2](https://apex.ipk-gatersleben.de/apex/f?p=284:45:::NO::P45_GENE_NAME:HORVU2Hr1G072850.2) | [chr2H](http://plants.ensembl.org/Hordeum_vulgare/Location/View?r=chr2H:524812874-524818497) | 524812874 | 524818497 | HC_G | Cell division protein FtsZ |
| [HORVU2Hr1G072880.2](https://apex.ipk-gatersleben.de/apex/f?p=284:45:::NO::P45_GENE_NAME:HORVU2Hr1G072880.2) | [chr2H](http://plants.ensembl.org/Hordeum_vulgare/Location/View?r=chr2H:525243771-525244507) | 525243771 | 525244507 | HC_G | ferredoxin 3 |
| [HORVU2Hr1G072890.6](https://apex.ipk-gatersleben.de/apex/f?p=284:45:::NO::P45_GENE_NAME:HORVU2Hr1G072890.6) | [chr2H](http://plants.ensembl.org/Hordeum_vulgare/Location/View?r=chr2H:525246625-525250587) | 525246625 | 525250587 | HC_G | Glucan endo-1,3-beta-glucosidase 14 |
| [HORVU2Hr1G072920.1](https://apex.ipk-gatersleben.de/apex/f?p=284:45:::NO::P45_GENE_NAME:HORVU2Hr1G072920.1) | [chr2H](http://plants.ensembl.org/Hordeum_vulgare/Location/View?r=chr2H:525333661-525335022) | 525333661 | 525335022 | HC_G | Senescence-associated protein, putative |
| [HORVU2Hr1G072930.3](https://apex.ipk-gatersleben.de/apex/f?p=284:45:::NO::P45_GENE_NAME:HORVU2Hr1G072930.3) | [chr2H](http://plants.ensembl.org/Hordeum_vulgare/Location/View?r=chr2H:525339860-525343114) | 525339860 | 525343114 | HC_G | RRNA intron-encoded homing endonuclease |
| [HORVU2Hr1G072950.1](https://apex.ipk-gatersleben.de/apex/f?p=284:45:::NO::P45_GENE_NAME:HORVU2Hr1G072950.1) | [chr2H](http://plants.ensembl.org/Hordeum_vulgare/Location/View?r=chr2H:525342060-525342445) | 525342060 | 525342445 | HC_G | Tar1p |
| [HORVU2Hr1G072960.1](https://apex.ipk-gatersleben.de/apex/f?p=284:45:::NO::P45_GENE_NAME:HORVU2Hr1G072960.1) | [chr2H](http://plants.ensembl.org/Hordeum_vulgare/Location/View?r=chr2H:525348646-525351998) | 525348646 | 525351998 | HC_G | Senescence-associated protein |
| [HORVU2Hr1G072990.1](https://apex.ipk-gatersleben.de/apex/f?p=284:45:::NO::P45_GENE_NAME:HORVU2Hr1G072990.1) | [chr2H](http://plants.ensembl.org/Hordeum_vulgare/Location/View?r=chr2H:525353082-525353628) | 525353082 | 525353628 | HC_G | RRNA intron-encoded homing endonuclease |
| [HORVU2Hr1G073030.6](https://apex.ipk-gatersleben.de/apex/f?p=284:45:::NO::P45_GENE_NAME:HORVU2Hr1G073030.6) | [chr2H](http://plants.ensembl.org/Hordeum_vulgare/Location/View?r=chr2H:525481435-525483107) | 525481435 | 525483107 | HC_G | Carbonic anhydrase |

**Supplemental Table 4.** Description of 31 different *mat-c* mutants including parental origin, year of isolation, treatment, special characteristic and Nordic Gene Bank (NGB) reference number. The Barley Genetic Stock (BGS) reference number for *mat-c* mutants is BGS 579 (www.nordgen.org/bgs).

| **Name** | **Parental cultivar** | **Isolation year** | **Treatment** | **Additional characteristic** | **NGB number** |
| --- | --- | --- | --- | --- | --- |
| *mat-c.16* | Bonus | 1956 | neutrons | short spike | 110016 |
| *mat-c.19* | Bonus | 1957 | neutrons | short spike | 110019 |
| *mat-c.32* | Bonus | 1958 | ethylene imine | - | 110032 |
| *mat-c.93* | Bonus | 1979 | ethyl methanesulfonate | short spike | 110093 |
| *mat-c.94* | Bonus | 1979 | ethyl methanesulfonate | short spike | 110094 |
| *mat-c.758* | Bonus | 1975 | neutrons | - | 110758 |
| *mat-c.760* | Bonus | 1975 | ethyl methanesulfonate | - | 110760 |
| *mat-c.770* | Bonus | 1975 | sodium azide | short culm | 110770 |
| *mat-c.865* | Bonus | 1965 | sodium azide | semi-sterility | 110865 |
| *mat-c.881* | Bonus | 1979 | sodium azide | laxatum spike | 110881 |
| *mat-c.907* | Bonus | 1980 | sodium azide | - | 116862 |
| *mat-c.910* | Bonus | 1980 | sodium azide | - | 117441 |
| *mat-c.913* | Bonus | 1982 | sodium azide | - | 117444 |
| *mat-c.926* | Bonus | 1982 | sodium azide | - | 117457 |
| *mat-c.943* | Bonus | 1982 | sodium azide | globosum grain | 117474 |
| *mat-c.101* | Foma | 1959 | ethylene imine | - | 110101 |
| *mat-c.122* | Foma | 1959 | neutrons | short spike | 110122 |
| *mat-c.400* | Foma | 1967 | ethylene imine | - | 110400 |
| *mat-c.1096* | Frida | 1985 | X-rays | - | 117627 |
| *mat-c.1107* | Frida | 1985 | X-rays | short spike | 119555 |
| *mat-c.1108* | Frida | 1985 | X-rays | short spike | 119556 |
| *mat-c.1109* | Frida | 1985 | X-rays | short spike | 119557 |
| *mat-c.1111* | Frida | 1985 | sodium azide | short spike | 119559 |
| *mat-c.1114* | Frida | 1985 | sodium azide | late in maturity | 119562 |
| *mat-c.1115* | Frida | 1985 | sodium azide | erect growth habit | 119563 |
| *mat-c.745* | Kristina | 1974 | iso-propyl methanesulfonate | - | 110745 |
| *mat-c.1102* | Semira | 1985 | neutrons | very short spike | 117633 |
| *mat-c.1118* | Semira | 1985 | X-rays | short spike | 119566 |
| *mat-c.1120* | Semira | 1985 | neutrons | short spike | 119568 |
| *mat-c.966* | Sv 79353 | 1982 | sodium azide | - | 117497 |
| *mat-c.1091* | Sv Ög 74233 | 1984 | sodium azide | - | 117622 |
